# *Orientia tsutsugamushi:* comprehensive analysis of the mobilome of a highly fragmented and repetitive genome reveals the capacity for ongoing lateral gene transfer in an obligate intracellular bacterium

**DOI:** 10.1128/msphere.00268-23

**Published:** 2023-10-18

**Authors:** Suparat Giengkam, Chitrasak Kullapanich, Jantana Wongsantichon, Haley E. Adcox, Joseph J. Gillespie, Jeanne Salje

**Affiliations:** 1 Mahidol-Oxford Tropical Medicine Research Unit, Faculty of Tropical Medicine, Mahidol University, Bangkok, Thailand; 2 Department of Microbiology and Immunology, Virginia Commonwealth University Medical Center, School of Medicine, Richmond, Virginia, USA; 3 Department of Microbiology and Immunology, School of Medicine, University of Maryland Baltimore, Baltimore, Maryland, USA; 4 Department of Pathology, University of Cambridge, Cambridge, United Kingdom; 5 Department of Biochemistry, University of Cambridge, Cambridge, United Kingdom; 6 Cambridge Institute for Medical Research, University of Cambridge, Cambridge, United Kingdom; The University of Texas Medical Branch at Galveston, Galveston, Texas, USA

**Keywords:** *Orientia tsutsugamushi*, Rickettsiales, obligate intracellular bacteria, intracellular pathogens, mobile genetic elements, comparative genomics, gene transfer agents, integrative and conjugative elements, lateral gene transfer

## Abstract

The rickettsial human pathogen *Orientia tsutsugamushi* (Ot) is an obligate intracellular Gram-negative bacterium with one of the most highly fragmented and repetitive genomes of any organism. Around 50% of its ~2.3-Mb genome is composed of repetitive DNA that is derived from the highly proliferated Rickettsiales amplified genetic element (RAGE). RAGE is an integrative and conjugative element (ICE) that is present in a single Ot genome in up to 93 copies, most of which are partially or heavily degraded. In this report, we analyzed RAGEs in eight fully sequenced Ot genomes and manually curated and re-annotated all RAGE-associated genes, including those encoding DNA mobilization proteins, P-type (*vir*), and F-type (*tra*) type IV secretion system (T4SS) components, ankyrin repeat- and tetratricopeptide repeat-containing effectors, and other piggybacking cargo. Originally, the heavily degraded Ot RAGEs have led to speculation that they are remnants of historical ICEs that are no longer active. Our analysis, however, identified two Ot genomes harboring one or more intact RAGEs with complete *F-T4SS* genes essential for mediating ICE DNA transfer. As similar ICEs have been identified in unrelated rickettsial species, we assert that RAGEs may play an ongoing role in lateral gene transfer within the Rickettsiales. We also identified a conserved set of gene transfer agent genes in all Ot genomes. Together these findings indicate that, despite their obligate intracellular lifestyle and host range restricted to mites, rodents, and humans, Ot genomes are highly dynamic and shaped through ongoing invasions by mobile genetic elements and virus-like elements.

**IMPORTANCE:**

Obligate intracellular bacteria, or those only capable of growth inside other living cells, have limited opportunities for horizontal gene transfer with other microbes due to their isolated replicative niche. The human pathogen Ot, an obligate intracellular bacterium causing scrub typhus, encodes an unusually high copy number of a ~40 gene mobile genetic element that typically facilitates genetic transfer across microbes. This proliferated element is heavily degraded in Ot and previously assumed to be inactive. Here, we conducted a detailed analysis of this element in eight Ot strains and discovered two strains with at least one intact copy. This implies that the element is still capable of moving across Ot populations and suggests that the genome of this bacterium may be even more dynamic than previously appreciated. Our work raises questions about intracellular microbial evolution and sounds an alarm for gene-based efforts focused on diagnosing and combatting scrub typhus.

## INTRODUCTION

*Orientia tsutsugamushi* (Ot) is an obligate intracellular Gram-negative bacterium that is a symbiont of trombiculid mites and causes the vector-borne human disease scrub typhus. Ot is a member of the alphaproteobacterial order Rickettsiales, which contains three well-studied families: Anaplasmataceae, Rickettsiaceae, and Midichloriaceae (1, 2), as well as four lesser-known families that have recently been described (Deianiraeaceae, Mitibacteraceae, Gamibacteraceae, and Athabascaceae) (3–5). As a lineage within Rickettsiaceae, genus *Orientia* also includes *Candidatus Orientia chiloensis*, which has recently been identified as an endemic species in Chile (6), and *Candidatus Orientia chuto*, which was isolated from a patient in Dubai (7). There is extensive strain diversity within the Ot species, which can be found in rodents, mites, and human patients across Southeast Asia. While strain diversity corresponds to differences in virulence in patients and in animal infection models, the molecular basis of these differences in virulence is not well understood. Ot strains are often classified according to serotype groupings, which are organized based on the human serological response to the highly antigenic surface protein TSA56. Major serotype groups are named after type strains and include Karp, Kato, Gilliam, Japanese-Gilliam, TA763, Saitama, Kuroki, Kawasaki, and Shimokoshi.

At around 2.0–2.5 Mb, the genome of Ot is almost double the size of most Rickettsiales genomes and is one of the most fragmented and repetitive bacterial genomes reported to date (8–10). With almost 50% of the genome composed of repetitive DNA sequences, many experimental approaches are challenging, i.e., primer design, gene and genome sequencing, gene prediction and annotation, and comparative genomics. Complete genome sequences of two strains, Boryong and Ikeda, were published in 2008 using short-read sequencing and bacterial artificial chromosome cloning (8, 9). Six additional strains (Karp, Kato, Gilliam, TA686, UT76, and UT176) were fully sequenced in 2018 using long-read PacBio technology (11). A comparison of these eight genomes enabled the identification of 657 core genes and an open pangenome that is heavily characterized by gene duplication and pseudogenization rather than the import of novel genes (11). Of these eight strains for which complete genome sequences are available, all except TA686 are pathogenic and were isolated from human patients (11). TA686 was isolated from a tree shrew (*Tupaia glis*).

The Ot genome is dominated by an integrative and conjugative element (ICE) called Rickettsiales amplified genetic element (RAGE) (8, 9, 12) that has proliferated rampantly throughout the genome and is present in over 70 copies. RAGEs encode numerous repeated and pseudogenized genes, as well as single-copy cargo genes that appear to be important for bacterial growth and pathogenesis. While the high number of RAGE copies in the Ot genome remains unmatched, similar RAGEs have been described in several other *Rickettsia* species: *Rickettsia bellii* (RBE) (single intact copy) (13), *Rickettsia buchneri* (seven complete or near-complete genomic copies and two plasmid encoded copies) (12), *Rickettsia massiliensis* (single intact copy (14), *Rickettsia parkeri* str. Atlantic Rainforest (single intact copy) (15), *Rickettsia felis* str. LSU-Lb (one plasmid encoded copy) (16), and *Rickettsia peacockii* (one partially degraded copy) (17). In Ot, the ICE has not been controlled by the bacterial host, and the RAGE has replicated to high levels in the genome (8, 9, 11). The reasons for the differential fate of the RAGEs in Rickettsiaceae are unknown, but the small effective population size as well as the presence of population bottlenecks in obligate intracellular bacteria likely explain why it has had the ability to proliferate without strong negative selection in at least some rickettsial species.

Here, we present a thorough Ot phylogenomics analysis and re-annotation of the RAGEs and their cargo genes in eight strains: Gilliam, Boryong, UT76, UT176, Karp, Kato, Ikeda, and TA686 (Table 1). We delineate the start and stop sites of all the intact and degraded RAGEs, allowing us to identify inter-RAGE (IR) regions with conserved clusters of genes. We further describe complete RAGEs in two Ot genomes (Kato and Gilliam). Finally, we annotate and classify the genes associated with DNA mobilizations and divergent type IV secretion systems (T4SSs), as well as the numerous multicopy cargo genes within the RAGEs, including those encoding ankyrin-repeat containing proteins (Anks) and tetratricopeptide repeat containing proteins (TPRs), which are putative secreted effectors. This detailed disentangling of the superfluous RAGE-dominated mobilome from the core and accessory Ot genome is expected to enlighten research on Ot biology and overall genome evolution in obligate intracellular bacteria.

**TABLE 1:** Accession numbers of genomes used in this study

Strains	Genome accession numbers	Links
Boryong	AM494475.1	https://www.ncbi.nlm.nih.gov/nuccore/AM494475.1
	NC009488.1	https://www.ncbi.nlm.nih.gov/nuccore/NC_009488.1
UT76	LS398552.1	https://www.ncbi.nlm.nih.gov/nuccore/LS398552
UT176	LS398547.1	https://www.ncbi.nlm.nih.gov/nuccore/LS398547.1
Karp	LS398548.1	https://www.ncbi.nlm.nih.gov/nuccore/LS398548.1
Kato	LS398550.1	https://www.ncbi.nlm.nih.gov/nuccore/LS398550.1
Ikeda	AP008981.1	https://www.ncbi.nlm.nih.gov/nuccore/AP008981.1
	NC_010793.1	https://www.ncbi.nlm.nih.gov/nuccore/NC_010793.1
TA686	LS398549.1	https://www.ncbi.nlm.nih.gov/nuccore/LS398549.1
Gilliam	LS398551.1	https://www.ncbi.nlm.nih.gov/nuccore/LS398551.1

## RESULTS AND DISCUSSION

### RAGE and IR regions

The RAGE is an ICE that is present in Ot and certain other Rickettsiales genomes. The degree of amplification and degradation of RAGE in the Ot genome is so extensive—making up around 50% of the Ot genome in 71–93 distinct genomic regions—that the beginning and end sites of RAGEs cannot be easily identified by visual inspection. Here, we established objective criteria for the classification of genes into Ot RAGEs and manually delineated each RAGE in the eight Ot strains in our study.

First, one or more copies of each of the following mobilization genes must be present: integrase (*int*), transposase, and F-type T4SS genes/relaxosome (*tra/trb*). Second, genes encoding one or more previously defined cargo or regulation proteins (9) must be present: membrane proteins (reclassified here as Ot_RAGE_membrane protein, see below), DNA adenine methyltransferase (*dam*), DNA helicase, ATP-binding proteins (*mrp*), histidine kinases (HKs), SpoT-related proteins (synthetase and/or hydrolase domains), HNH endonuclease, peroxiredoxin, Anks, and TPRs. Third, the RAGE region begins with the first RAGE-associated gene and continues until a previously defined core gene (11) or another RAGE is reached. A run of core genes is classified as an IR element. The identification of boundaries between RAGEs is described in Materials and Methods. Fourth, given the abundance of genes encoding hypothetical proteins (HPs) within RAGEs, those located between mobilization/cargo RAGE genes are classified as being part of that RAGE. However, HP-encoding genes located between RAGE mobilization/cargo genes and Ot core genes that cannot be resolved as being within either RAGE or IR regions are classified as isolated HP-encoding genes. Fifth and final, single or multiple mobilization or cargo RAGE genes are classified as isolated mobile genes or cargo genes, respectively. Some new cargo genes were identified by virtue of residing within RAGE regions in most or all genomes, and these are discussed below.

**Fig 1:**
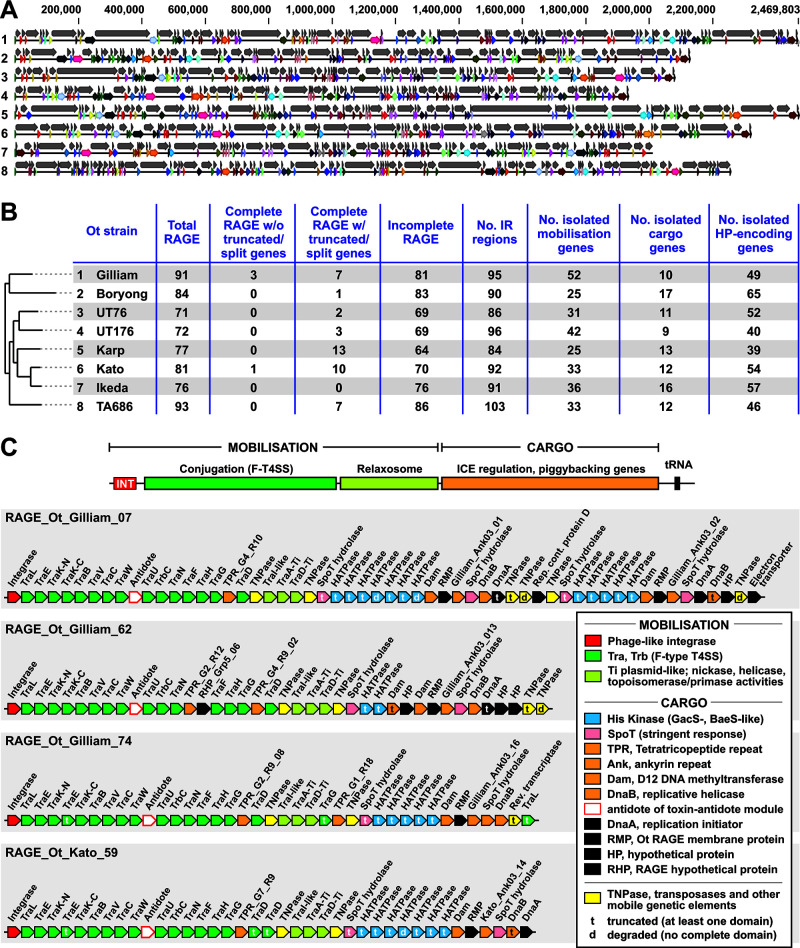
Ot RAGE and IR elements. (A) An overview of the genomes of eight Ot strains with genes classified into RAGE and IR regions. Numbers at the left refer to Ot strains listed in panel B. Gray arrows indicate RAGE regions; colored arrows denote IR regions. The colors correspond to conserved IR regions between strains and demonstrate the lack of synteny between Ot genomes. (B) Table summarizing RAGEs, IR regions and isolated mobile genes, cargo genes, and hypothetical genes that could not be classified into RAGE or IR elements. Ot strains are listed accordingly to a previously estimated phylogeny (11), with numbers corresponding to full genome maps in panel A. (C) Organization of genes in the four complete RAGEs found in our analysis. Detailed analysis of the re-annotation and classification of all genes in the eight genomes are given in data set S1. d, degraded (no identifiable domains present); t, truncated (at least one identifiable domain present).

In this way, the entire genome of each Ot strain was classified into the following regions: RAGE region, IR region, isolated HP-encoding gene, isolated mobilization gene, and isolated cargo gene (data set S1). Using these criteria, we identified 71–93 RAGEs in the eight analyzed genomes (Fig. 1A and B). The patterns of RAGE fragmentation and pseudogenization varied extensively between strains, and it was not possible to map RAGEs between strains (Fig. 1A). This implies that RAGEs entered Ot strains one or more times as intact elements and subsequently underwent replication, pseudogenization, and recombination in independent trajectories.

Most RAGEs in Ot are degraded, both in terms of either completely lacking RAGE-associated genes or retaining genes that have been truncated or fragmented into predicted pseudogenes. A previous study found that the Ot strain Ikeda genome lacked any complete RAGES (9). We assessed whether any of the strains in our analysis encoded complete RAGEs, defined as containing a full set of mobilization genes and additional cargo genes as outlined in previous analyses (9). All the strains in our analysis, with the notable single exception of Ikeda, encoded one or more complete sets of RAGE genes (Fig. 1B). However, most of those RAGEs contained one or more mobilization genes that were truncated. Accordingly, we carried out sequence alignments to define each RAGE gene as being full length, truncated (containing one or more identifiable domains) or degraded (containing no identifiable complete domains). We then assessed whether any strains contained complete RAGEs with intact, full-length genes (Fig. 1B). We found that two strains, Gilliam and Kato, encoded complete RAGEs with full-length mobilization genes (Fig. 1C). This suggests that these strains may have obtained these elements recently and that they may be capable of continued mobilization. ICEs normally have a preferred integration site, often within tRNA genes (12), as observed for *Rickettsia* species (18). Despite our discovery of complete RAGEs in these Ot genomes, no identifiable integration sites could be determined.

We previously used RNA sequencing analysis and comparative genomics to show that, despite the lack of synteny between Ot strains driven by the prolific RAGEs, small groups of proximate genes were transcribed at similar levels and maintained synteny across strains (19). This demonstrates selection for gene order at the local level despite it being absent at a global level across Ot genomes. To further identify gene groups evolving under strong selective constraints relative to superfluous RAGEs, we analyzed the IR regions, which harbor most core Ot genes (Fig. 1A). This revealed 84–103 IR regions, ranging in length from 2 to 27 genes (Fig. 1B). The difference in number of IRs between genomes is accounted for by some IR gene blocks being split up in some genomes and not others, rather than the presence of novel genes in some genomes. Identification of these conserved IR gene groups illuminates highly conserved microsynteny that may encompass functionally linked genes sharing expression and/or regulatory programs.

### Single-copy cargo genes

**Fig 2:**
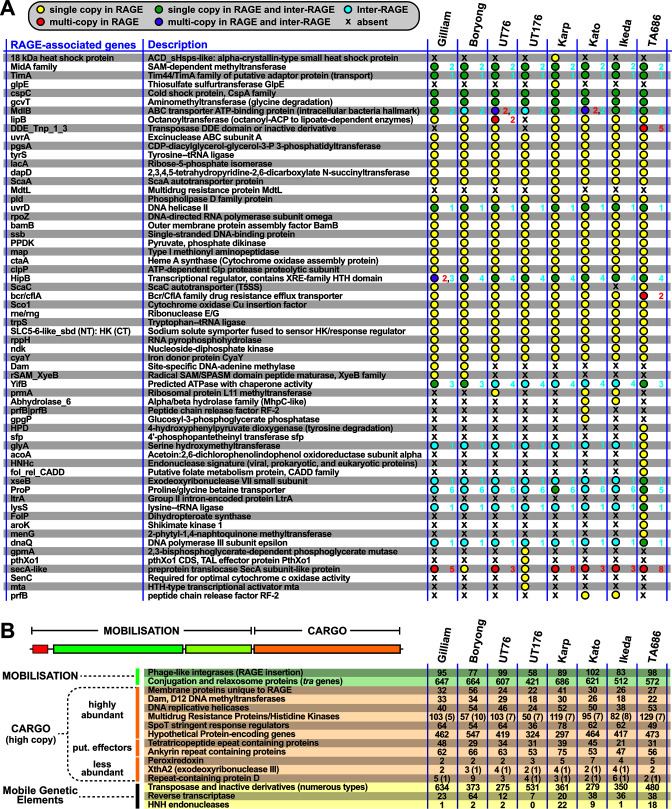
Single and multi-copy cargo genes encoded on Ot RAGEs. (A) Single or low-copy cargo genes encoded on Ot RAGE. Summary statistics show whether genes are present in single or multiple copies on RAGEs in different strains, and also in single or multiple copies in IRs. The exact number of copies is given for each gene. Blue text indicates the number of copies in IR; red text denotes the number of copies in RAGE. (B) Frequency and distribution of high copy cargo genes (both full length and truncated/degraded) within RAGEs in eight strains of Ot. Numbers in brackets denote additional copies in IRs.

The delineation of Ot genomes into RAGE and IR regions enabled us to better characterize RAGE cargo genes (Fig. 2A and B). In addition to the group of highly replicated multicopy cargo genes already described as RAGE components (discussed below), we identified numerous single-copy genes previously overlooked for their occurrence within RAGEs (Fig. 2A). These include genes involved in fundamental processes of bacterial physiology and metabolism, e.g., tyrosine tRNA ligase, RNA polymerase subunit omega, and the ClpP protease, as well as genes encoding predicted secretory effectors likely involved in interactions with host cells, including phospholipase D and autotransporter proteins ScaA and ScaC in all genomes, and ScaB (Boryong), ScaF (TA686) and ScaG (TA686) in individual strains. As many of these single-copy genes have orthologs in other bacterial species that lack the RAGEs, it is likely that they were not introduced by mobile genetic elements. Rather, their current presence within RAGEs indicates they were probably incorporated into RAGE via recombination. However, a case-by-case analysis may reveal certain conserved genes shuttling between Ot genomes via RAGE mobilization. For instance, despite their conservation in all *Rickettsia* genomes, genes encoding secreted effectors and metabolite transporters were previously found piggybacking on RAGEs in the *R. buchneri* genome, illustrating the ability of RAGE to shuttle rickettsial genes important for the obligate intracellular lifestyle (12).

### Highly abundant multi-copy cargo genes

Analysis of RAGE-associated cargo genes revealed 16 genes or gene groups present in numerous copies in all eight Ot genomes (Fig. 2B). Gene groups included those encoding membrane proteins, Dam DNA methyltransferases, DNA helicases, multi-drug resistance proteins (MRPs) and histidine kinases, SpoT hydrolase and synthetases, hypothetical/uncharacterized proteins, mobile genetic elements (i.e., insertion sequences (ISs), transposases, integrases, and reverse transcriptases), Anks, TPRs, and Vir- and Tra-type T4SS proteins. All of these, except *vir-*type T4SS genes, have been identified as RAGE cargo genes in previous studies (8, 9, 18, 20). For each gene group, we assessed (i) whether all the genes annotated as belonging to this category were paralogs of the same gene or whether multiple distinct genes were present within one group, and (ii) whether some or all genes within a group were truncated and not able to form a full-length protein and, where a functional domain was known, whether this domain was present or not. Genes involved in DNA mobilization, effector proteins, and T4SS genes are discussed in dedicated sections below, while other multi-copy cargo genes are discussed here.

#### Membrane proteins

**Fig 3:**
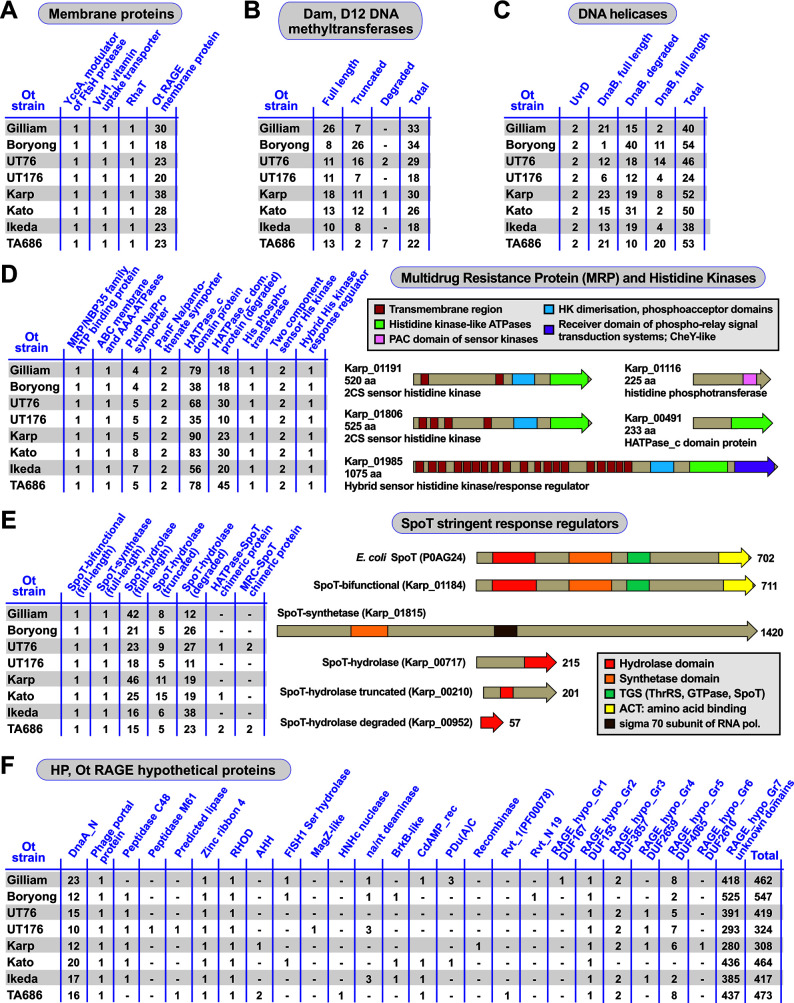
Analysis of high copy cargo genes on RAGE elements in Ot. (A–C) Frequency and distribution of RAGE cargo genes annotated as (**A**) membrane proteins, (**B**) Dam DNA methyltransferases, and (**C**) DNA helicases. DnaB is a replicative DNA helicase, and UvrB is a repair DNA helicase. (D) Frequency and distribution of RAGE cargo genes encoding MRP/histidine kinases, with examples of His kinase divergent architectures. (E) Frequency and distribution of RAGE cargo genes encoding SpoT stringent response regulators, with examples of divergent architectures. The bifunctional SpoT protein is compared to the canonical SpoT protein of *Escherichia coli*. (F) Frequency and distribution of RAGE cargo genes encoding HPs. AHH, adenosyl homocysteine hydrolase; BrkB-like, YihY/virulence factor BrkB family protein; CdAMP_rec, cyclic diAMP receptor proteins; DUF, domain of unknown function; DnaA_N, N-terminal domain of DnaA; MagZ, nucleoside triphosphate pyrophospho-hydrolase; na/nt, nucleic acid/nucleotide deaminase; PDu(A)C, copper chaperone; RHOD, rhodanese homology domain; Rvt_1 (PF00078), reverse transcriptase Pfam PF00078; Rvt_N 19, domain of reverse transcriptase Rvt_N.

The eight Ot genomes encode 21–41 RAGE-associated genes annotated as membrane proteins (Fig. 3A, data set S2). Analyses revealed that each Ot strain encodes exactly one copy of three genes encoding proteins with analogy to characterized membrane proteins: the YccA modulator of protease FtsH, vitamin transporter Vut1, and a gene similar to the rhamnose transporter RhaT. The remaining 18–38 genes encode paralogs of a gene we call Ot_RAGE_membrane protein, ranging in length from 90 to 663 bp. This protein lacks homology to any non-Ot genes, and no known domain could be identified. Thus, the function of this gene in Ot is unknown.

#### DNA methyltransferases

Ot genomes encode 18–34 genes with similarity to *dam*, all of which are located within RAGEs (Fig. 3B). Sequence alignments demonstrate that 8–26 encode full-length proteins, defined as being equal in length to *Escherichia coli* (ECO) Dam and encoding all seven known domains. The Ot genomes encode an additional 2–26 truncated *dam* genes where some domains are preserved and fewer degraded copies with no identifiable domains. It is not known if these genes are functional, although previous studies (19, 21) showed that they were not detected by proteomics analysis. They may have a specific role in methylation of RAGE during mobilization and/or integration to protect from deleterious effects of single-stranded DNase activity.

#### DNA helicases

DNA helicases unwind double-stranded DNA and function in DNA and RNA metabolism, with general roles in DNA replication, repair, and recombination. We found that all strains of Ot encode exactly two full-length copies of the DNA helicase *uvrD*, which is involved in DNA repair (Fig. 3C). One copy is located within a RAGE, while the second is located within IR82. The *dnaB* family of helicases, by contrast, is more numerous and degraded in Ot genomes (Fig. 3C). This helicase is involved in DNA replication and is present in 36–52 copies, with 1–23 being full length and mostly located within RAGEs. Each genome encodes one full-length copy located at the interface of IR46 and IR47, which is likely the ancestral non-RAGE paralog involved in genome replication. Other copies are undoubtedly associated with RAGE mobilization. Our previous proteomics analysis (19, 21) detected expression of a *dnaB* gene, but due to sequence similarities between numerous paralogs, it was not possible to determine which specific gene was expressed. Given its role in DNA replication, however, it is expected that at least one paralog would be expressed and functional.

#### MRPs and histidine kinases

We identified around 100 genes in the Ot genomes that were annotated as MRPs or histidine kinases (Fig. 2B and 3D; Fig. S1; data set S3) or were found to contain histidine kinase domains (e.g., the sodium/proline symporter *putP*). MRPs are members of the ATP-binding cassette (ABC) transporter protein family and were annotated as MRPs based on the presence of a histidine kinase ATPase domain (HATPase). Our analysis determined that genes encoding two “MRP” proteins were distinct from all the other HATPase domain containing proteins in Ot: an MRP/NBP35 family ATP-binding protein and an ABC-membrane and AAA ATPase protein, both single copy in each genome. The former was located within an IR region (IR9) and the latter within RAGEs. Analysis of the remaining genes annotated as MRP/histidine kinases led us to identify several full-length orthologs of two-component system (2CS) histidine kinase genes. These include one histidine phosphotransferase gene (which does not contain an HATPase domain), one large hybrid sensor histidine kinase/response regulator gene, and two 2CS histidine kinase genes (Fig. 3D). All were present in the same copy number and locations in each genome. Histidine phosphotransferase and the two 2CS sensor histidine kinase genes were consistently found in IR regions IR43, IR47, and IR63, respectively, while the hybrid sensor histidine kinase/response regulator gene was located within a RAGE. In our UT76 proteomics data set (21), both the histidine phosphotransferase and the hybrid sensor histidine kinase/response regulator genes were detected, while the two sensor histidine kinase proteins were not (data set S1). We also identified two copies of a histidine kinase domain containing sodium/pantothenate symporter, *panF*, present in IR11 and IR55 regions in each genome, as well as a sodium/proline symporter, *putP*, present in four to eight copies and distributed into both RAGEs and IRs. Analysis of our previous proteomics data set showed that *panF* was expressed in strain UT76 as was one copy of *putP* located in IR49 (21) (data set S1). The remaining MRP/histidine kinase genes were paralogs of one another, containing an HATPase domain and being present in 45–113 copies per genome with various degrees of truncation (Fig. S1). We classified these as degraded HATPase domain containing proteins when an intact HATPase domain could no longer be detected due to the short length of the gene (Fig. 3D; Fig. S1; data sets S1 and S3).

#### SpoT stringent response regulators

SpoT is a bifunctional synthetase/hydrolase that is essential for inducing and regulating the stringent response in *E. coli* and other bacteria through mediating inter-cellular levels of (p)ppGpp, also called alarmone (22, 23). Ot genomes encode 36–78 genes with homology to *spoT*. We identified exactly one full-length *spoT* gene, present in an IR region (IR46), encoding both synthetase and hydrolase domains in all Ot genomes (Fig. 3E). This gene was the only *spoT* homolog found to be expressed in our previous proteomics analysis and was shown to be upregulated in extracellular Ot, consistent with a role in transitioning between different bacterial states (21). We also identified exactly one gene in each genome that encodes only the *spoT* synthetase domain in addition to a long C-terminal domain of unknown function. We then identified 15–46 *spoT* genes that lacked the synthetase domain yet encoded the intact hydrolase domain as well as a further 16–44 *spoT* genes that were truncated or degraded such that a functional hydrolase domain was no longer present. Finally, we identified one to four genes in some genomes in which hydrolase domains are fused to other genes. Most rickettsial genomes harbor 6–12 *spoT* genes, with some of the abovementioned architectures present (data not shown). Curiously, bifunctional (complete hydrolase and synthetase domains) genes are typical of most other Rickettsiales species, though not common in *Rickettsia* species and absent from notable human pathogens (e.g., *Rickettsia prowazekii*, *Rickettsia typhi*, *Rickettsia rickettsii*, and *Rickettsia conorii*) (12). Still, the tendency for all Rickettsiales genomes to retain numerous single-domain *spoT* genes, even when RAGEs are absent, implies their function in some aspect of the stringent response. The presence of such drastic numbers and diverse architectures of *spoT* genes in Ot genomes relative to other rickettsial species is intriguing and deserving of future investigation.

#### HPs

The Ot strains harbor 308–547 genes per genome that are annotated as hypothetical or uncharacterized, of which about half are located within RAGEs (Fig. 2B and 3F; data set S4). We determined whether all the RAGE hypothetical/uncharacterized genes were paralogs of a single RAGE gene or if they encoded multiple different genes. Sequence alignments for all the genes annotated as hypothetical or uncharacterized in the Karp genome were performed, which revealed that the genes clustered into 24 groups (Fig. 3F) with 18 of these encoding genes carrying known protein domains. Those without known domains were named RAGE_hypo_Gr1-7, with groups I–VI encoding a domain of unknown function and group VII combining a mixture of all remaining HP genes with no identifiable known domains. Three of these genes with known domains were present in exactly one copy in all genomes and encode a phage portal protein, a zinc ribbon domain, and a rhodanese homology domain. Another single-copy gene found in all eight Ot genomes carries a domain of unknown function (DUF155). Furthermore, hypothetical genes containing a *dnaA* N-terminal domain were identified in 10–23 copies in all Ot genomes. In our prior study, one full-length paralog, located in IR1 was expressed in UT76, while others were not detected (21) (data set S1).

Other hypothetical/uncharacterized genes were distributed sporadically among the genomes. In order to get a sense of the distribution of the remaining RAGE-associated hypothetical genes that were not clustered into conserved groups, we analyzed the remaining hypothetical genes in the RAGEs of the Karp genome only. There were no identifiable domains in any of these and the diversity was such that it was not possible to bin them into homologous groups. We annotated them all as belonging to a large and divergent 25th group (RAGE_hypo_Gr7). While little can be inferred about the function of these hundreds of genes, it is likely that at least some of these play important roles in the biology of Ot.

### Putative effectors piggybacking on Ot RAGE

#### Anks

The ankyrin repeat (AR) is one of the most common protein folds in nature, being widespread in eukaryotes and pervasive in many viruses and host-associated bacteria (24, 25). Ankyrin repeats are used to mediate a myriad of protein-protein interactions, and host-associated prokaryotes and viruses frequently express Anks to hijack or subvert host cell pathways that would be detrimental or beneficial to their survival (26). Previously, several Ot Anks were shown to be secreted via the rickettsial type I secretion system (27). Certain Ank effectors have been functionally characterized in strain Ikeda and shown to play important roles in host cell interactions (27–31). However, a major challenge in comparing the host-pathogen cell biology of different Ot strains has been the difficulty assessing which Anks are most similar to those in other strains. This is important for determining the significance of Anks as species-specific versus strain-specific effectors underlying pathogenesis.

**Fig 4:**
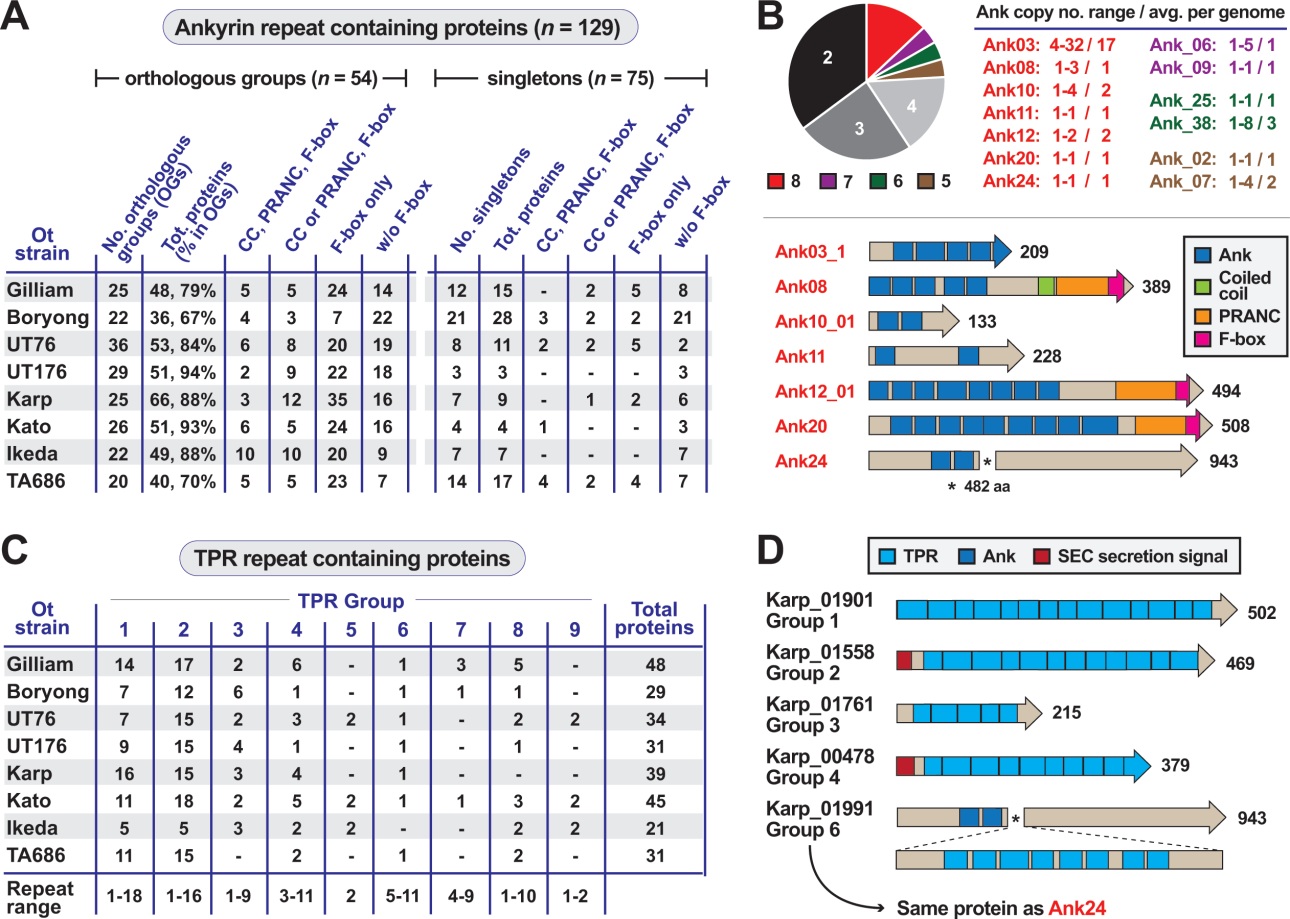
Putative effectors piggybacking on Ot RAGE. (A) Frequency and distribution of Anks in Ot genomes. Anks are broken down into orthologous groups (OGs, present in two or more genomes) or singletons (unique to a genome). CC, coiled coil; PRANC domain found at the C terminus of certain pox virus proteins; F-box, motif of approximately 50 amino acids that functions in protein-protein interactions. (B) (Top) Graphical view of Ank OG strain representation (two to eight genomes). Roughly 25% of Ank OGs are found in five or more strains, with variable levels of conservation in copy number per genome. (Bottom) Architectures for Anks present in all Ot genomes, with proteins from Ot strain Karp. (C) Frequency and distribution of TPRs in Ot genomes. Nine ortholog groups contain all the TPRs across eight Ot genomes. (D) Examples of diverse TPR architectures for six proteins from Ot strain Karp. PRANC, pox protein repeats of ankyrin, C-terminal.

We defined a set of criteria for clustering Anks, with their subsequent characterization within each Ot genome following the well-described Ank repertoire of strain Ikeda (9). We identified several new Ank groups in Ikeda, although some of these lack complete Ank repeats and are likely non-functional (data set S5). Our comparative analysis indicates Ot strains encode 47–66 Anks, with variability (67%–94%) in the number of common versus strain-specific genes per genome (Fig. 4A). Ot Anks often harbor a single F-box domain, which are prominently known components of SCF (Skp1, Cullin1, and F-box) ubiquitin ligase complexes, but recent studies have described their participation in non-SCF protein-protein interactions involved in diverse eukaryotic pathways (32). F-box-resembling pox protein repeats of ankyrin, C-terminal (PRANC) domains and coiled coils were less frequently predicted.

Of the 54 orthologous groups of Ot Anks, all genomes were found to encode at least one copy of seven groups: *ank03, ank08, ank10, ank11, ank12, ank20*, and *ank24* (Fig. 4B). *ank03* is by far the most prominent Ank, being present in 4–32 copies in Ot genomes, with the other six families present in 1–4 copies (Fig. 4B). Curiously, while most Ot Anks are predominantly found within RAGEs, *ank20* is encoded in an IR region (IR84) in all analyzed Ot genomes. Collectively, these seven Anks likely carry out essential functions in Ot biology. However, each strain likely utilizes unique Ank arsenals throughout its life cycle, given that some of the less conserved Anks have characterized functional roles; e.g., Ank01 and Ank06 of Ot str. Ikeda modulate NF-kB transport to the nucleus (30). As such, it is likely that there is functional redundancy between the Ank groups, with some of the 100 other Ank groups not found in Ikeda functioning similarly to *ank01* and *ank06* in genomes lacking these genes.

#### TPRs

The tetratricopeptide repeat is another protein motif that is commonly used in mediating inter-protein interactions, typically found in subunits of multi-protein complexes (33). TPR repeat containing proteins are widespread in Ot (Fig. 4C), albeit with a lower number of copies per genome than Anks. Ot TPRs have been less characterized than the Ot Anks, with only one report demonstrating a role in inhibition of eukaryotic translation in Ot strain Boryong (34). We compiled the 21–48 TPRs per Ot genome and classified them into nine groups primarily based on within-protein location of tetratricopeptide repeats (Fig. 4C; Fig. S2; data set S5). While the positions were conserved within groups, the number of repeats was variable and indicated expansion and contraction of repeats, as well as processive gene degradation within each group (Fig. 4D). The prediction of SEC signal peptides in certain TPRs indicates at least some of these putative effectors may be secreted to the periplasm with possible translocation across the outer membrane, possibly via TolC as proposed for the RARP-1 effector of *R. typhi*. (35) Still, the lack of N-terminal secretion signals in most TPRs indicates other possible routes for TPR secretion that await characterization.

### Mobile genetic elements associated with RAGE

#### Integrases

**Fig 5:**
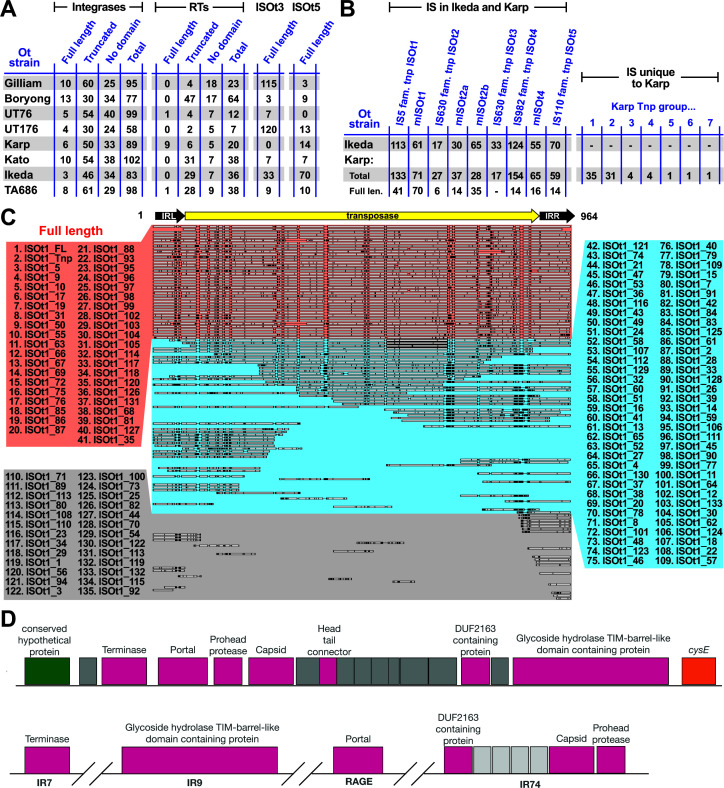
A diversity of mobile genetic elements associates with Ot RAGEs. (A) Frequency and distribution of Ot RAGE-associated genes encoding integrases, group II intron-associated reverse transcriptases, and IS elements ISOt3 and ISOt5. (B) Frequency and distribution of IS elements in Ikeda and Karp strains. (C) Alignment showing classification of ISOt1 elements as full length or degraded. Full-length copies of ISOt1 in Karp are shown by the red dotted box. (D) Overview of GTAs in Ot compared with the canonical GTA cluster in *Rhodobacter capsulatus*. GTA genes conserved between Ot and *R. capsulatus* are shown in magenta. GTA genes in *R. capsulatus* that are not found in Ot are shown in dark gray. Ot genes positioned between GTA genes are shown in light gray. Figure adapted from Lang and Beatty (37). GTA, gene transfer agent.

ICEs, such as Ot RAGE, encode integrase genes to catalyze genomic integration and conjugative genes (discussed in the next section) to catalyze horizontal gene transfer (36). The Ot genomes analyzed in this study encode 58–102 integrase genes (Fig. 5A), of which only 3–13 per genome remain full length, consistent with progressive degradation of the Ot RAGE. Where present, the integrases are located at the start position of a RAGE (Fig. 1C). However, several integrase genes were located as isolated genes outside RAGE regions, reflecting the high mobility of these genes and the overall high recombination rates in Ot genomes.

#### Transposable elements

In addition to the OtRAGE, the Ot genome encodes two other types of transposable elements (9): “copy and paste” retrotransposons (group II introns) and “cut and paste” DNA transposons. While these are independent mobile genetic elements, they have been incorporated into the Ot RAGE regions, and it is likely that the different mobilizable elements impact each other’s activity. Group II introns are self-splicing retrotransposons that catalyze their integration into genomes via an RNA intermediate using an intron-encoded reverse transcriptase protein (38). The Ot genomes encode 7–64 group II intron reverse transcriptase genes, although only UT76, Karp, and TA686 encode full-length genes, with the others being heavily degraded (Fig. 5A). All full-length reverse transcriptase genes were immediately followed by an HNH endonuclease gene likely required for catalysis.

Ot encodes several families of DNA transposons which have been previously classified in strain Ikeda (9). This class of mobile elements is composed of a transposase gene flanked by inverted repeat (IR) regions on either side, which together make up an insertion sequence (IS) (39). Numerous families of IS have been identified in other bacteria. Given the large number of IS elements in each Ot genome and their highly degrative tendency, we selected two of the many frequently occurring IS genes, ISOt3 and ISOt5, to characterize in detail across all eight genomes (Fig. 5A). These were present in 0–120 (ISOt3) and 0–70 (ISOt5) full-length copies across the genomes. We also analyzed the complete set of IS elements in one strain, Karp, and compared these with those previously predicted for str. Ikeda (Fig. 5B). An example of the analysis of one IS element in Karp, ISOt1, shows the distribution of full-length and degraded copies typical of IS families in all Ot genomes (Fig. 5C). We identified the same set of IS elements that had previously been described in Ikeda (9) (Fig. 5B and C). We followed the classification and nomenclature established in Nakayama et al. (9) in which mISOt1, mISOt2 and mISOt4 denotes “miniature” versions of elements containing the same terminal inverted repeat sequences as ISOt1, ISOt2, and ISOt4, respectively. Within the nine IS classes found in Karp, most were heavily degraded with some, such as IS630 family transposase ISOt3, having no remaining full-length elements. We identified seven additional groups of transposase genes in Karp that were not part of IS elements (Fig. 5B).

#### Gene transfer agents

In exploring the different machineries that might carry out lateral gene transfer in Ot, we searched for evidence of genes that might have originated from phages. We identified phage-like genes such as that encoding capsid protein in all the Ot genomes in our analysis. Upon closer inspection, these were found to closely resemble the recently described gene transfer agents (GTAs) of *Wolbachia* (40). This is a DNA element that includes several virus-like genes but is distinct from bacteriophages. First described and best characterized in *Rhodobacter capsulatus*, this genetic element packages genomic DNA rather than viral DNA and does not cause lysis in recipient cells. GTAs were previously shown to be widespread in alphaproteobacteria, including Rickettsiales (37). We found that all the Ot genomes carry six GTA genes encoding terminase, glycoside hydrolase TIM-barrel-like domain containing protein, portal, DUF2163 containing protein, capsid, and the prohead protease (Fig. 5D; data set S7). All except the portal protein have conserved positions in IRs 7, 9, and 74 in all genomes, while portal is always encoded on a RAGE. The conserved nature of this set of genes in all Ot genomes is highly suggestive of an ongoing role for GTAs, although its activity and potential role in lateral gene transfer remain to be characterized experimentally.

### Type IV secretion systems

#### F-T4SS

**Fig 6:**
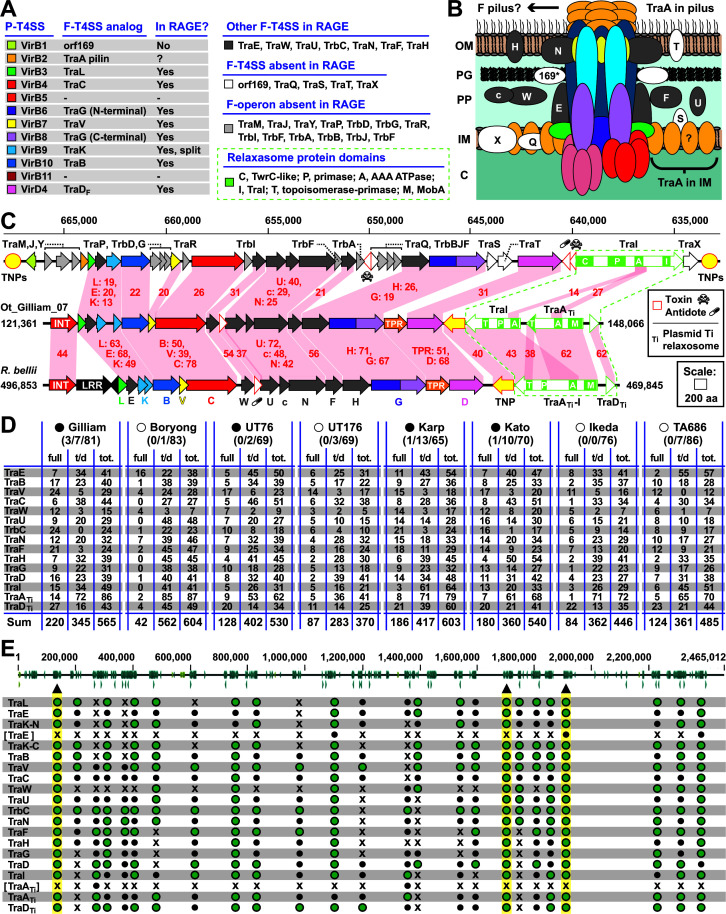
Characteristics of the F-type T4SS and relaxosome proteins encoded on Ot RAGEs. (A) Composition of the Ot RAGE F-T4SS in relation to the *Agrobacterium tumefaciens vir* P-T4SS and the *Escherichia coli tra/trb* F-T4SS from the F operon. Analogs across divergent T4SSs are colored similarly, with other colors as follows: dark gray, RAGE T4SS proteins found in F-T4SSs but not P-T4SSs; white, *E. coli* F-T4SS scaffold genes not present in RAGE T4SSs; light gray, other *E. coli* F operon genes not present in RAGE. For relaxosome proteins (olive green), domains were predicted with SMART (44). (B) Theoretical assembly of the RAGE T4SS in relation to data from other F- and P-type T4SSs. The uncertain synthesis of a pilus is depicted (see text for details). (C) Comparison of the *E. coli* F operon to mobilization genes of complete RAGEs from Ot str. Gilliam and *Rickettsia bellii* str. RML369-C. This *E. coli* strain, K-12 ER3466 (CP010442), has the F operon on a chromosomal segment flanked by transposases (yellow circles). Red shading and numbers indicate % aa identity across pairwise alignments. Dashed lines enclose the relaxosome genes, whose protein domains are described in panel A. (D) Frequency and distribution of of full-length and truncated *tra*/*trb* genes in Ot strains. Complete circles, genomes containing full sets of *tra*/*trb* genes within one or more RAGE; open circles, no complete *tra*/*trb* gene sets. Numbers in parentheses: number of complete RAGEs/number of complete RAGE genes containing truncated genes/incomplete RAGEs. Details of truncated genes and gene fusions are given in data sets S1 and S8. (E) Genomic location of *tra*/*trb* gene clusters in Ot str. Gilliam. Triangles and highlighting depict complete RAGEs. Bracketed TraE and TraA_TI_ are commonly occurring pseudogenized duplications. Green circles, complete gene; small black circles, predicted pseudogene; Xs, gene absent with *tra*/*trb* gene cluster. INT, integrase; LRR, leucine rich repeat protein.

The RAGE encodes a conjugative T4SS highly similar to the F-T4SS of the archetypal F plasmid of *E. coli* (*tra/trb*) (8, 9). Previous comparisons of the RAGE T4SS with that of the *E. coli* F plasmid showed that it encodes 14 proteins predicted to form the T4SS scaffold, some of which are analogous to components within P-type T4SSs (18, 41) (Fig. 6A and B; data set S8). While syntenic to the *E. coli tra*/*trb* T4SS, the RAGE T4SS lacks genes involved in the regulation of conjugation, as well as other assembly factors and lytic transglycosylases (Fig. 6C). In this way, the RAGE T4SS is a streamlined version of the canonical F-T4SS. The Ot RAGE T4SS is also highly similar in gene order and composition to F-T4SSs characterized in the RAGEs of *R. buchneri* (12), *R. bellii* (13), *R. felis (*16), and *Rickettsia massiliae*. (14) As with these prior reports, we also did not identify a gene encoding a pilin protein (typically TraA in F-T4SSs) in RAGE T4SSs, though it may be that a pilus is synthesized using a different pilin gene since the RAGE-harboring *R. bellii* forms large pili during host infection (13). Experiments are needed to determine if the RAGE T4SS builds a pilus or functions pilus-less, as is noted for the P-T4SS of *Rickettsia* species (41), *Neorickettsia risticii* (42), and likely all Rickettsiales (43). Another common peculiarity of these F-T4SSs is the split gene encoding TraK, the significance of which is unknown.

The relaxosome of the *E. coli tra*/*trb* F-T4SS encodes one multi-functional relaxase, TraI, which excises and binds single-stranded plasmid DNA (45). In contrast, the Ot RAGE carries three genes, *traI*, *traA_Ti_*, and *traD_Ti_*, predicted to comprise the relaxosome that mobilizes RAGE (Fig. 6C). *E. coli* TraI harbors four distinct domains required for nicking, binding, and unwinding DNA. By contrast, Ot TraI lacks a domain for nicking DNA and shares very limited similarity to *E. coli* TraI. However, Ot TraA_Ti_ carries a MobA-like domain that cleaves single- and double-stranded DNA at specific sites (46). Curiously, all the domains encompassed by Ot TraI and TraA_Ti_ proteins are found in a single *Rickettsia* RAGE protein named TraA_Ti_-I, which is highly similar to both Ot TraI and TraA_Ti_ but shares limited similarity to *E. coli* TraI. As their annotation indicates, RAGE TraA_Ti_ and TraD_Ti_ are similar to relaxosome proteins of plasmid Ti of *Agrobacterium tumefaciens* (ATU), TraA and TraD, which are required for T-DNA translocation into plant cells via the *vir* T4SS (47). The significance of different relaxosome structures between Ot and *Rickettsia* RAGEs is unclear, although *traA_Ti_* and *traD_Ti_* genes are common on *Rickettsia* plasmids even when RAGEs are absent (48). It may be that multiple RAGE types exist in the rickettsial mobilome and are defined by their cognate relaxosomes. The presence of transposases flanking relaxosome genes in all complete Ot and *Rickettsia* RAGEs may also signify that RAGEs evolve by recombining different relaxosome cassettes into the conjugation and cargo genes.

Over 50% of the RAGE T4SS and relaxosome genes are present as truncated pseudogenes, and some of these clusters encode only a subset of the 18 possible Ot RAGE mobilization genes (Fig. 6D and E). Given the high degree of pseudogenization, we sought to examine whether any strain encoded any RAGE mobilization gene clusters containing a complete complement of full-length genes. We found that Karp, Kato, Gilliam, and UT76 encoded at least one complete RAGE mobilization gene set, while Ikeda, Boryong, TA686, and UT176 did not (Fig. 6D and E; Fig. S3; data set S7). There was a positive correlation between strains containing complete sets of RAGE mobilization gene sets and the total number of full-length mobilization genes (Fig. 6D and E). Moreover, the complete RAGE mobilization gene clusters in Gilliam and Kato were located within complete RAGE regions (Fig. 1C and 6E; Fig. S3). While several genomes lack complete RAGE mobilization gene clusters, all except Boryong encode at least one full-length copy of each RAGE mobilization gene, albeit not in a contiguous cluster. Therefore, it is possible that all strains except Boryong could assemble a functional F-type T4SS competent to mediate transfer of RAGEs.

#### P-T4SS

**Fig 7:**
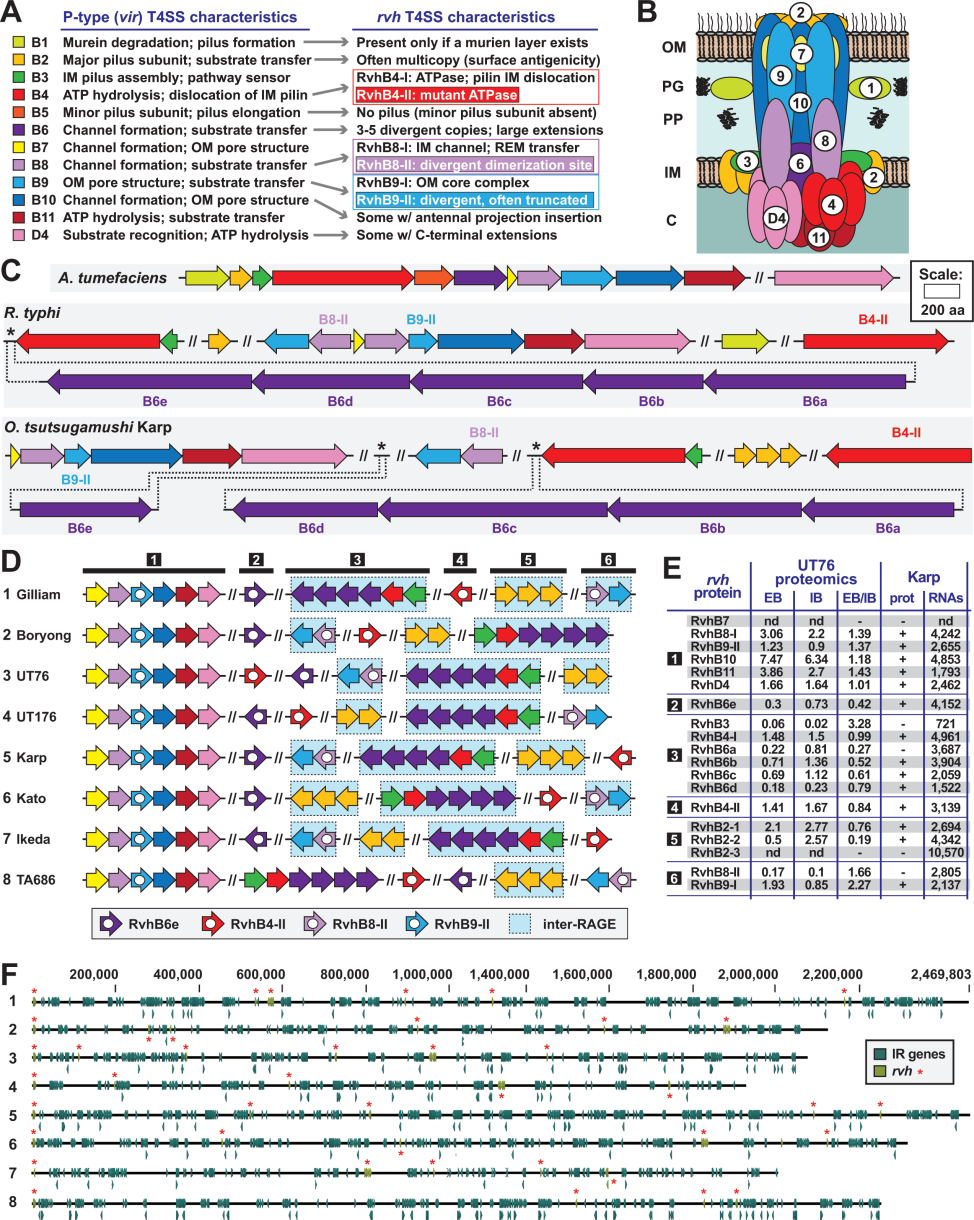
Synopsis of Ot P-type (*vir*-like) T4SS genes. (A) Description of the general *rvh* T4SS characteristics, summarized from prior studies (41, 43, 49, 50). (B) Theoretical assembly of the RAGE T4SS in relation to data from other P-type T4SSs. There is no synthesis of a T-pilus (see text for details). (C) Comparison of genes encoding *vir* T4SS in *Agrobacterium tumefaciens*, the archetypal P-T4SS, and those encoding the *rvh* T4SS in *Rickettsia typhi* and Ot. (D) Arrangement of *rvh* genes in Ot genomes. Red genes are located in RAGE regions, while blue are located in IR regions. (E) Previously published RNAseq and proteomics data showing relative expression levels of *rvh* genes in strains UT76 and Karp. These are taken from Atwal et al. (UT76) (21) and Mika-Gospodorz et al. (19) (Karp). UT76 data show relative peptide counts in IB and EB. Karp data show presence or absence of detectable peptides from proteomics analysis (±) and relative RNA transcripts from RNAseq data (TPM). (F) Distribution of *vir* genes across Ot genomes showing lack of conservation of absolute position, despite similarities in gene groupings as shown in Fig. 7D. EB, extracellular bacteria; IB, intracellular bacteria; TPM, transcripts per million.

Like other rickettsial species, Ot encodes a P-type T4SS related to the archetypal *vir* T4SS of the pTi plasmid of *A. tumefacians*. Thus, Ot encodes a second class of T4SS in addition to the RAGE-encoded F-T4SS. Relative to *vir*, this Rickettsiales *vir* homolog (*rvh*) T4SS has distinct features, including the scattered distribution of *rvh* gene clusters; duplication of *rvhB8*, *rvhB9*, and *rvhB4*; three to five copies of *rvhB6*; and no gene encoding an equivalent to the VirB5 minor pilin subunit (43, 49, 50) (Fig. 7A and B). These characteristics are nuanced: (i) RvhB4-II, RvhB8-II, and RvhB9-II carry atypical structural deviations from described VirB4, VirB8, and VirB9 family proteins; (ii) RvhB6 proteins have large insertions flanking the VirB6-like membrane spanning region; and (iii) a lack of a minor pilin subunit precludes formation of a T-pilus (50, 51). There is evidence that structurally different RvhB8-I and RvhB8-II proteins of *R*. typhi cannot dimerize (51), which leads to the hypothesis that divergent duplications may autoregulate effector secretion (50). The recent identification of *rvh* genes in all seven Rickettsiales families implies a highly important function (3, 52). Thus, we assessed the properties of the Ot *rvh* T4SS in the face of its rampant mobile genetic element-induced genome shuffling.

A single set of *rvh* genes is present in all the Ot genomes analyzed here and has features resembling those described in other Rickettsiales species (Fig. 6A through C). Interestingly, Ot shares two key characteristics with the *rvh* T4SS of distantly related Anaplasmataceae species as opposed to that of closely related *Rickettsia* species. First, while Ot lacks genes for a VirB5 protein and therefore cannot form extracellular pili used for cellular attachment, it encodes two to three copies of *rvhB2*, the major pilus subunit. Given that Ot lacks lipopolysaccharide (LPS) on its surface, multiple RvhB2 proteins may act as divergent surface antigens in a similar fashion previously posited for Anaplasmataceae species, which collectively lack LPS biosynthesis genes and have multiple *rvhB2* genes throughout their genomes (43, 53). Second, Ot lacks any identifiable *rvhB1* gene, which is present only in *Rickettsia* spp. This gene encodes a lytic transglycosylase predicted to cleave peptidoglycan (PGN) to allow T4SS scaffold assembly (49). Compared with *Rickettsia* spp., which synthesize a canonical PGN layer (54, 55), the presence of a minimal cell wall in Ot and Anaplasmataceae species is consistent with the absence of *rvhB1* from these genomes (56, 57). These collective differences in *rvh* T4SS architecture present clear convergent evolution in Ot and Anaplasmataceae species in the context of shared cell wall morphology and probable responses to host cell immune pressures (58).

Our analysis shows that the identities of six *rvh* gene clusters are conserved across Ot strains, while the genomic positions of the clusters vary between strains (Fig. 7D). Clusters 1 (*rvhB7*, *rvhB8-I*, *rvhB9-II*, *rvhB10*, *rvhB11*, and *rvhD4*), 2 (*rvhB6e*), and 4 (*rvhB4-II*) are located within RAGEs in all strains, with the other *rvh* genes consistently located in IR regions except for cluster 6 and clusters 3 and 6 in UT176 and TA686, respectively (Fig. 7D and F). Analysis of published data sets of proteomics and RNAseq data in Karp and UT76 (19, 21) show that RvhB2-3 and RvhB7 proteins are not detected under growth conditions used in those analyses, although transcription levels of *rvhB2-3* are high (Fig. 7E). All the other Rvh proteins are detected in UT76, and most are detected in Karp. The UT76 data set compared peptide levels in two different bacteria populations: intracellular bacteria (IB) and extracellular bacteria (EB) (21). The EB:IB ratio of some multi-copy Rvh proteins differs between paralogs, e.g., RvhB2-1, which is present at a ratio of 0.76 compared with the ratio of 0.19 for RvhB2-2. This suggests expression of these proteins may be differentially regulated, potentially reflecting functional differences. Our collective analyses indicate that Ot Rvh genes can form a functional P-T4SS, despite the pervasive mobile element-induced gene shuffling in Ot genomes. While no Ot *rvh* transported effector has been described to date, it is highly likely that Ot utilizes the *rvh* T4SS during host cell infection, as secreted proteins that interact with the T4SS gatekeeper, RvhD4, have been described for *R. typhi* (59–61), *R. rickettsii* (60, 62), *Anaplasma marginale* (63), *Anaplasma phagocytophilum* (64–70) and *Ehrlichia chaffeensis* (66, 71–74).

### Ot lacks defense mechanisms against invasive DNA

We show here that the Ot genome is exceptional in its abundance of invasive mobile genetic elements, including ICEs, transposases, group II introns, and GTAs. Bacteria have evolved a range of anti-viral mechanisms to minimize damage caused by mobile genetic elements (75–77). We therefore sought to assess if Ot lacks these protective systems, possibly explaining the invasion and subsequent proliferation of mobile genetic elements. We used DefenseFinder to carry out a systematic search for all known antiphage systems including restriction modification systems, CRISPr/Cas systems, and toxin-antidote defense modules (76, 78). We found that none of the Ot strains in our study had any identifiable defense systems. While it is possible that this is due to sequence divergence, small size (e.g., certain toxin-antidote modules), or systems that have not yet been discovered, the software was able to detect three different restriction modification systems and the newly described Pyscar defense system (79) in the closely related free-living alphaproteobacterial *Caulobacter crescentus* (CCS). In addition to lacking identifiable anti-viral defense systems, Ot also has limited homologous recombination capability, a system that is frequently used in anti-viral defense (76). While Ot encodes RecA and the alternative homologous recombination pathway RecFOR, it lacks the major repair complex RecBCD that can defend against some mobile genetic elements by degrading linear double-stranded DNA (11). Overall, Ot lacks identifiable mobile genetic element defense systems likely explaining the proliferation of mobile DNA in these genomes.

### Conclusions

The identification of complete RAGEs in two Ot strains raises the possibility that these ICEs remain actively mobilizable both within and between Ot genomes. Evidence for this hypothesis awaits the sequencing of intact and complete genomes of large numbers of Ot isolates beyond the small number currently available. While only two genomes encode complete RAGEs with full length genes, all encode all the genes required for RAGE mobilization, albeit in dispersed locations across the genome. Future research is needed to determine whether such mosaic RAGEs can be mobilized or not.

The identification of potentially active RAGEs in Ot raises the question of how they can be transferred between Ot organisms during their life cycle. Ot is an obligate intracellular bacterium, and therefore, bacterial cells have limited interactions with other bacteria of the same or different species. It has been shown that simultaneous infection with multiple Ot strains is found during human infection (80), and it is therefore possible that different strains of Ot can infect the exact same host cell in a mite or a rodent during co-infection by two species. Albeit rare, this could occur with sufficient frequency to enable horizontal gene transfer between species. Alternatively, it is possible that the extracellular form of Ot retains sufficient residual metabolic activity to support lateral DNA transfer in the cell-free extracellular state. Mites typically feed in a tight cluster, for example, on the ear of an infested rodent, and the co-feeding pool may provide the environment for close encounters between Ot cells in an intracellular or extracellular state to mediate conjugation.

In conclusion, this study has led to the manual re-annotation of the genomes of eight strains of Ot, enabling the delineation of RAGE and IR regions. Open questions remain. Importantly, while intact RAGEs have been identified in two strains, the dynamics of the Ot RAGE are completely unknown. It is also unknown whether the current set of RAGEs within one genome results from one or multiple invasion events. The Ot RAGE encodes an F-T4SS, but it is not known if these are active, nor what they transport beyond the ICE itself. Progress toward answering these questions will enable further insights into the biology and pathogenicity of this important human pathogen.

## MATERIALS AND METHODS

### Identification of RAGE and inter-RAGE regions in the genome of Ot

The boundaries of RAGE and IRs were manually delineated in each genome using defined criteria. First, groups of genes whose positions relative to one another were conserved across strains were identified manually by comparing the genomes of eight Ot strains (Ikeda, Boryong, Karp, Kato, Gilliam, TA686, UT76, and UT176). This led to the identification and numbering of IR regions. RAGE regions were subsequently identified using criteria largely drawn from Nakayama et al. (9).

The element was classified as a “complete RAGE” if the element encoded a full-length integrase gene at the left end (N-terminus), a full-length transposase gene, full-length set of conjugative transfer genes (tra genes: *traA*, *traB*, *traC*, *traD*, *traE*, *traF*, *traG*, *traH*, *traI*, *traK*, *traL*, *traN*, *traU*, *traV*, and *traW*), and non-conjugative genes (RAGE-associated cargo genes) including one or all of the following: SpoT-related proteins [ppGpp hydrolase, (p)ppGpp synthetase, SpoT synthase, and SpoT hydrolase], DNA methyltransferase, DNA helicase, histidine kinases, ATP-binding proteins (mrp), HNH endonuclease, membrane proteins, ankyrin repeat proteins, and hypothetical proteins. The RAGE-associated cargo genes in the “complete RAGE gene” can be either full-length or truncated genes.

The element was classified as a “complete RAGE with truncated genes” if the sequence encoded the same gene set as above, but where one or more of the integrase, transposase, or *Tra* conjugative transfer genes were truncated.

The element was classified as an “incomplete RAGE” if the sequence encoded integrases or transposases, and at least one RAGE-associated cargo gene.

Finally, we identified single genes, or groups of genes, that could not be classified as a RAGE or IR region using these criteria. The “isolated mobile gene” was defined as encoding one or more integrase or transposase without RAGE-associated cargo genes. The “isolated cargo gene” was defined as encoding one or more cargo genes without transposases, integrases, or *tra* genes. The “isolated hypothetical protein” was defined as encoding one or more hypothetical proteins at the boundary of conserved IRs or RAGEs.

**Fig 8:**
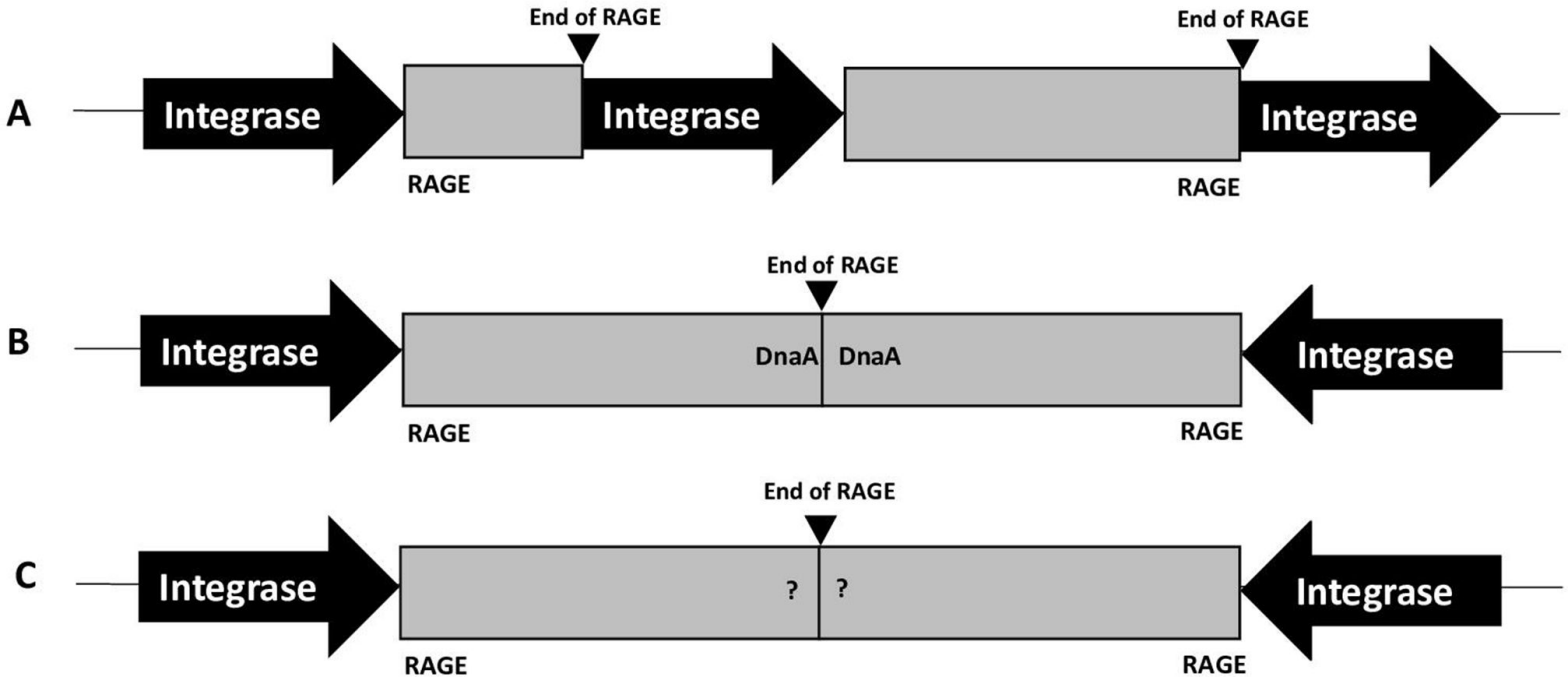
Identification of RAGE termination sites.

The presence of a *dnaA* gene was used as an indicator gene for defining the end of a RAGE element (Fig. 8). However, the criteria could not be applied for all RAGE elements when hypothetical proteins and transposases were located at the end of RAGE masking the original *dnaA* terminus. In the first case (Fig. 8A), RAGE elements are located next to each other in the same direction. In this case RAGE is terminated when the integrase gene of the next RAGE is found. In the second case (Fig. 8B), RAGE elements are located next to each other in opposite direction, and two *dnaA* genes are located next to each other. In this case, the RAGE is terminated at the *dnaA* gene, which belongs to RAGE on the left (forward direction) and RAGE on the right (reverse direction). In the third case (Fig. 8C), RAGE elements are located next to each other in opposite direction. Two RAGEs were combined into one RAGE if a *dnaA* gene was not present in either RAGE.

### Identification of RAGE-associated cargo genes

The list of conserved non-conjugative genes or RAGE-associated cargo genes were extracted based on Nakayama et al. (9). The sequences were then inspected manually by observing the length of genes, conserved motifs/domains, and other elements such as signal peptides and transmembrane regions. A gene was defined as a “full-length gene” if the sequence encoded a complete set of domains, a “truncated gene” if the sequence encoded only a partial set of domains and a “degraded gene” if no domain was identified on the sequence.

### Analysis of multi-copy cargo genes not associated with DNA mobilization

#### Membrane proteins

Membrane proteins were manually extracted from the genome database, and the SMART search engine (44) was used to identify membrane domains and other elements. A large group of genes with homology to each other, but no identifiable known domain, was renamed “OT_RAGE_membrane_protein.” The membrane protein encoding gene was assigned a new name if significant domains/main domains were found, such as autotransporter proteins (Sca family), vut1- putative vitamin uptake transporter, RhaT- permease of the drug/metabolite transporter superfamily, and Bax inhibitor-1 (BI-1)/YccA inhibitor of FtsH protease domains.

#### MRP/histidine kinases

**Fig 9:**
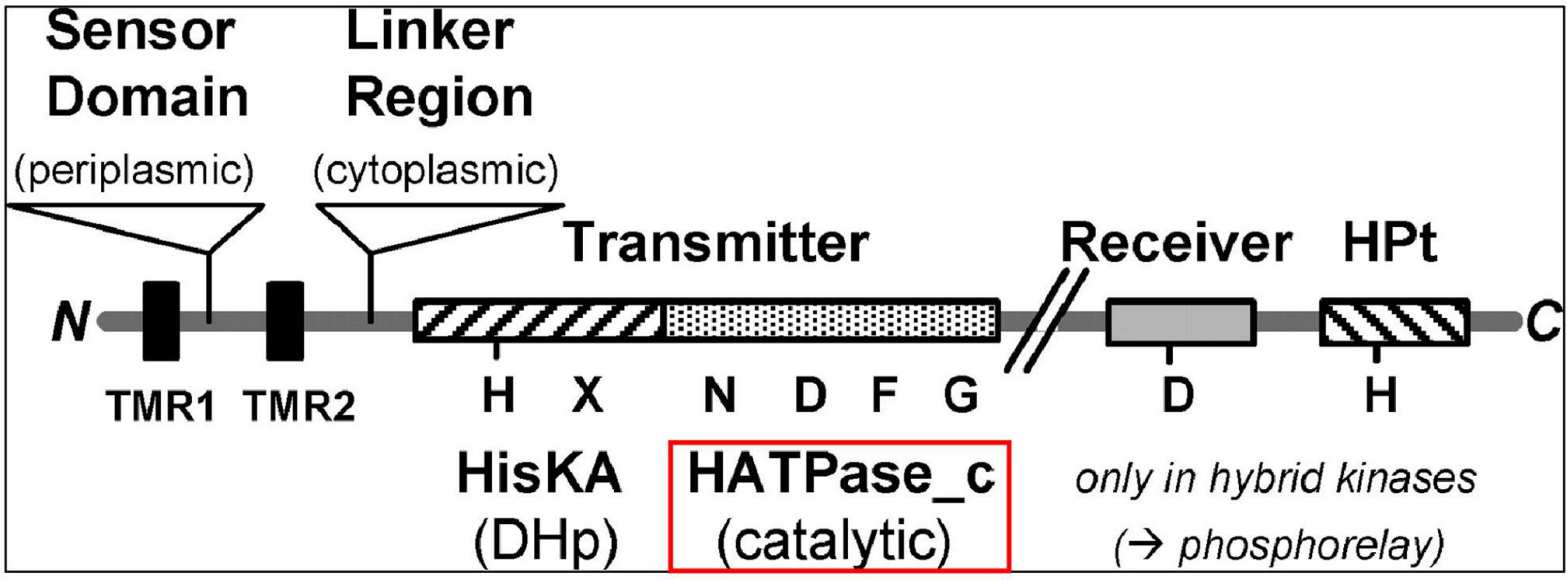
An overview of protein domains histidine protein kinases. Figure is modified from reference (84).

All genes previously annotated as HK and multi-drug resistance-associated proteins (MRPs) were extracted from the genome databases and analyzed using SMART (44). Both HK and mrp proteins contain an HATPase_c domain (histidine kinase-type ATPases catalytic domain) (Fig. 9). This domain is found in several ATP-binding proteins, including histidine kinase (81), DNA gyrase B (82), topoisomerases, and heat shock protein HSP90 (83). The HK and mrp genes were renamed based on the presence of an HATPase_c domain. HK or mrp proteins were renamed as “HATPase_c domain containing protein” if the search found an HATPase_c domain. HK or mrp proteins were renamed as “degraded HATpase_c” if no HATPase_c domains were found in the search. Where additional domains were identified, the genes were appropriately renamed, e.g., genes comprising two component sensor systems. Where additional domains were not identified, the HK and mrp proteins in this study were classified as truncated genes because they only contained the catalytic part (HATPase_c) and lacked other major domains such as sensor domain, HisKA (histidine kinase A domain dimerization/His phospotransfer), and receiver Hpt domain (histidine phosphotransfer) (Fig. 9) (84).

In addition, the same HATPase_c domain was also found in symporter proteins. The sodium:proline symporter “PutP” was classified as full length if the sequence contained symporter, HisKA, HATPase_c, and REC domains. PutP was was classified as “truncated PutP” or “degraded PutP” if the sequence lacked the symporter and REC domains. The symporter Sodium:pantothenate symporter “PanF” was classified as full-length if the sequence contained only the symporter region.

#### (p)ppGpp hydroplase/synthetases

We manually extracted all genes annotated as SpoT, (p)ppGpp, synthetase, and hydrolase from the genome databases. The sequences were then compared to their respective orthologs in ECO and CCS. Literature searches and GenomeNet motif search (pfam) were used to identify motifs and conserved regions in these sequences as shown (Table 2).

**TABLE 2:** Motifs conserved in SpoT^*a*^

Domains	Conserved	Motifs	References
HD domain (hydrolase)	Region	AIDYAIHYHGXQTRESGDPYYYHPLHVALIIAQMKXDTVSVITAL LHDTVEDTELTLSDIEREFGKEVAXLVDGVTKLXKLRFQSYHXQQ AXNFRKLLLAISNDIRVLLVKLADRLHNMRTIESIKLLNKRIRIALE TXEIYAPLAERIGA	(85)
	H1	HXXXXR/KXXG/QXXYXXXP/Q/WXX	(86)
	H2	I/VT/IAXLHD/N	(86)
	H3	XLLXKLXXRXHNXXXX	(86)
SYNTH domain (synthetase)	Region	G/ARXKXXYSIXXKMXXKXIXXXQLXDXXAXRXIXXXXXXXXXXC YXXLXXIHXXYXXXPXXXQDFIXXPKXNGYQSXHTXIXGPXXXXI EVQIRTXXMHXXXXXGXAAHWXYK	(85)
	G	GRHK	(86)
	YQS	NGYQSXHT	(87)
TGS domain (Thr-tRNA synthetase, GTPase, and SpoT domain)	Region	CFTPXGKLIALPKGATVVDFAYKXHSELGNKCIGAKISNKVVPLD TQLQNGDQVEIIT	(85)
	H	DFAYXXHXXXG	(88)
Helical domain	Region	TFAVTGKAQSEIRKFIRXQAYKKYIDLGKEILIQTLKKIQVANINV CIAKIAHXLNKKNVEEVFFXIGXELLSKKEIIKIIT	GenomeNet motif search (pfam)
CC domain (conserved cysteine/RIS-ribosome inter-subunit domain	CC	CCYPLPGDLIIGLCT	(85, 89)
ACT domain (aspartokinase, horismate mutase, and TyrA/RRM RNA recognition motif)	Region	RNKIGSLASITTILENNNXNICNIKTTNXTQSTXQIIIDIEISTLEQL NKIXNILQSSXDIISVXR	(85)

^*a*^ Underlined bases indicate important amino acids in the motif.

We employed a naming system based on the presence of domains/motifs in each CDS. Genes containing all domains (HD, SYNTH, TGS, helical, CC, and ACT) were annotated as SpoT. Genes having only the HD domain were annotated as SpoT hydrolase, while genes encoding only the SYNTH domain were annotated as SpoT synthetase. Genes encoding the HD domain but lacking one or more of the conserved histidines were annotated as truncated hydrolase. Short fragments that could be aligned to hydrolase but lack complete domains were annotated as degraded hydrolase. Genes containing HD domain merging with a part of HATPase were named as HATPase-SpoT hydrolase, and genes containing HD domain merging with a part of Mrp were named as Mrp-hydrolase, respectively.

#### DNA methyltransferases

DNA methyltransferase (MTase) genes were extracted from the genome and analyzed using SMART (44) for protein domain annotation. However, SMART does not provide the details of MTase motifs within the predicted domain. Therefore, multiple sequence alignments of DNA methyltransferase were carried out for identification of motifs using Geneious. We used the conserved amino acid residues in the Dam protein of *E. coli* (acc.no. P0AEE9) as a reference for identification of motifs I–VII and motif X of MTase at the C-terminal region (90). The protein sequence of DNA methyltransferase containing motifs I–VII and motif X was classified as a full-length gene. The protein sequence of DNA methyltransferase with incomplete motifs and unidentified motifs was indicated as truncated gene and degraded gene, respectively.

#### Replicative DNA helicases

DNA helicase genes were filtered from the genome, and their protein domains were characterized by SMART (44). Multiple sequence alignments of the helicase genes were then carried out in order to identify motifs. We used the conserved amino acid residues in the DnaB protein of *E. coli* K12 (accession no. NC000913.3) as a reference for the identification of motifs I–VII at the C-terminal region (91). The protein sequence of DnaB containing motifs I–VII was indicated as full-length gene. The protein sequence of DnaB with incomplete motif and unidentified motifs was indicated as truncated gene and degraded gene, respectively.

#### Uncharacterized proteins

Between 308 and 464 genes annotated as hypothetical or uncharacterized were found in the eight genomes of *Orientia*. In this study, we only manually analyzed uncharacterized proteins from Karp strain as a model to minimize the analysis time. Uncharacterized proteins from Karp were filtered from the genome, and the protein domain was characterized by SMART (44). Where clear groups of homologous genes within the set of Karp genes were found, these were classified into 25 defined groups. These were renamed according to known domains with which they had homology or named Ot_RAGE_hypo_group 1–7. These 25 groups were then aligned to the other seven genomes in our data set in order to determine the conservation of the groups of genes.

Some uncharacterized proteins were renamed and no longer classified as hypothetical proteins if they aligned to known genes such as DnaA, Phage portal protein, and Lipase3.

### Analysis of multi-copy genes involved in DNA mobilization

#### Insertion sequence transposable elements

The presence of IS elements in Ot was investigated using the online search tool ISfinder (92) to match with attributes and nomenclatures previously submitted for *Orientia*-specific IS (9). Each IS match was manually traced for completeness with flanking IRs and direct repeats along respective genome sequences. Extensive analysis was performed with Karp strain to identify the complete set of IS elements and classified into classes. Only ISOt3 and ISOt5 were systematically analyzed across all eight different Ot genomes.

#### Integrases

Genes annotated as integrase genes were extracted from the genomes, and protein domains were screened by SMART (44). Integrase in Ot is a phage integrase which is classified into two major families: the tyrosine recombinases and the serine recombinases, based on mode of catalysis (93). Multiple sequence alignment of phage integrase domain was analyzed for identification of motifs using Geneious. We used the conserved amino acid residues in Bacteriophage P2-integrase (accession no. AF063097.1) and Enterobacteria phage P2-integrase (accession no. NC_009488.1) as references for the identification of three domains; arm-type binding motifs at N-terminal region, core-type binding, and catalysis at C-terminal region. The His-X-X-Arg motifs and second conserved arginine on catalytic domain were also included in the alignment (93, 94). The protein sequence of phage integrase containing arm-type binding, core-type binding, and catalysis motifs was used to identify full-length genes. The protein sequence of phage integrase with incomplete motif and unidentified motif were annotated as truncated gene and degraded gene, respectively.

#### Reverse transcriptases

Reverse transcriptase (RVT) genes were filtered from the genome, and protein domains were characterized by SMART (44). Multiple sequence alignment of *rvt* was then analyzed for identificatiable motifs. We used the conserved amino acid residues of group II intron reverse transcriptase/maturase (LtrA) in *E. coli* (accession no. WP_096836589.1) and *Lactococcus lactis* (accession no. NZ_CP059048.1) as a reference for the identification of three domains: reverse transcriptases (RVT_N) at the N-terminal site, reverse transcriptases (RVT), and group II intron, maturase-specific domain (GIIM) at the C-terminal site (95–97). The protein sequence of reverse transcriptase containing RVT_N, RVT, and GIIM was used to identify full-length genes. Genes encoding an incomplete reverse transcriptase motif or no identifiable reverse transcriptase motif were classified as truncated and degraded genes, respectively.

#### Transposases

Genes annotated as transposase genes were first extracted from the genome. Then, protein domains and motifs were further characterized by SMART (44) and Geneious, respectively. Transposase genes in Ot belong to restriction endonuclease-like proteins or PD-(D/E)XK nucleases and DD[E/D]- transposase, which generally contain the catalytic domain and transposon-binding domain (98, 99). Some transposases additionally contain C-terminal or N-terminal domains (100). In this study, we used the conserved amino acid residues of in *E. coli* (accession no. NC 002695.2) as a reference for the identification of restriction endonuclease-like motifs (I–IV). Three conserved active sites in motifs II and III, one of which is aspartic acid (D), one is either glutamic (E) or aspartic acid (D) and/or the last one is lysine (K), were identified for characterization of PD-(D/E)XK nucleases and DD[E/D]- transposase motifs (98, 99). The protein sequence of transposase containing motifs (I–IV) and PD-(D/E)XK signature residues was used to identify full-length genes. Genes encoding an incomplete transposase motif or no identifiable motif were classified as truncated and degraded genes, respectively.

#### GTA genes

GTA genes in each of the Ot genomes were identified by BLASTp search using Wolbachia GTA genes (37, 40).

### Analysis of ankyrin repeat containing proteins

To identify AR proteins (Anks) from previously annotated records, all Ank sequences were extracted and analyzed using SMART (44) to predict Ank repeats and other domains including coiled coil, F-box, and PRANC. SMART (44) defines an Ank as a 33-residue motif. SMART predictions were confirmed by manual inspection and additional BLAST searches using Ank repeat domains. In some instances, additional Ank repeat domains were identified that were not detected by SMART. The method used to identify new Ank domains is given in data set S5.

Identification of homologous Ank repeats across Ot strains was carried out manually using Geneious. The criteria for identification of a homologous Ank was based on sequence similarity and repeated units. Individual Ank sequences of the other seven strains were blasted against Ot strain Ikeda. Then, the sequence that presented the highest identity (>80%–90%) was chosen to verify the similarity of ankyrin repeats and other domains. The sequences were given the name based on the published Ikeda Anks if the overall sequence’s identity to Ikeda Anks was more than 80% and presented a similar set of ankyrin repeats. The sequences were given a new name if the overall sequence identity to Ikeda Anks was less than 80% and/or presented a different set of ankyrin repeats; this included extra ankyrin repeat units or missing units.

**Fig 10:**
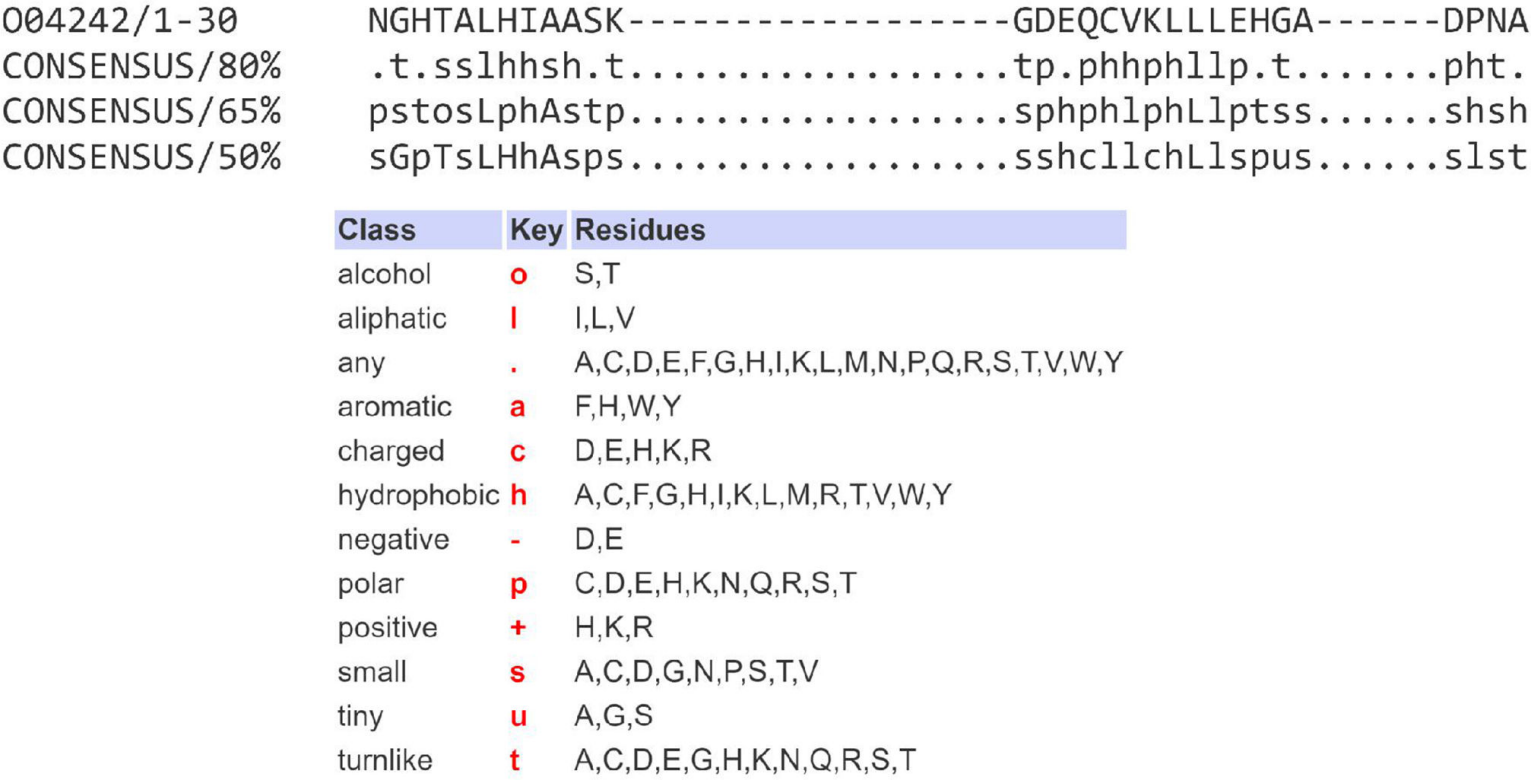
Consensus terms used by SMART for the identification of Ank domains.

Novel Anks or hypothetical proteins were manually identified and analyzed using Geneious. To identify additional Anks in Ikeda and the seven other strains in this study, each repeat unit of an individual published Ikeda Ank was imported into the “Find motifs” tool, and the maximum mismatches were set up to 10. Hits were verified using SMART. Then, the sequence of the novel Ank was BLASTed against the published Anks and within the strain to check whether it was different from previously identified Anks or not. The newly identified Anks were given a name by continued ranking after the published Ikeda Anks, starting with Ank21, Ank22, and Ank23 (Fig. 10).

### Analysis of tetratricopeptide repeat containing proteins

To identify TPR proteins from the previous annotated records of eight Ot strains, all TPR sequences were extracted and analyzed using SMART (44). The program predicted the location of TPR motifs and other domains including signal peptide and transmembrane region. All identified TPR proteins were grouped based on the similarity of the location of TPR motifs. Each group consists of one long gene, the master gene, and multiple shorter duplication remnants. Novel TPRs were identified by manual search using Geneious. All TPR proteins were renamed based on the group number followed the number of TPR repeats.

### Analysis of P-type IV secretion systems (*rvh*)

Literature search and BLAST search [National Center for Biotechnology Information (NCBI) and Kyoto Encyclopedia of Genes and Genomes (KEGG)] were performed to identify the presence of each Rvh subunit (RvhB1 to VirB11 and RvhD4) in Ot. The amino acid sequences of each Vir subunit present in Ot Boryong strain were compared to their respective orthologs in RBE and ATU for their percent of amino acids identity, length of amino acid sequences, and presence of motifs. The presence of motifs was used as the major criteria to identify Vir subunits as indicated in Table 3.

**TABLE 3:** Vir proteins and their motifs

Subunit(s)	Motif(s)	Reference(s)
VirB2-1	TM region 1 (GXXXXXXXXXXXXXXXIXXXXG), TM region 2 (AI**I/VI/V**XXX**A/S**XX)^*a*^	(101–103)
VirB2-2	TM region 1 (GXXXXXXXXXXXXXXXIXXXXG), TM region 2 (AI**I/VI**XXX**A/S**XX)^*b*^	
VirB2-3	TM region 1 (GXXXXXXXXXXXXXXXIXXXXG), TM region 2 (AI**I/VI**XXXSXX)	
VirB3	L-TRP-GV motif (LXXXXTRPXXXXGV)	(103, 104)
VirB4-1	Walker A (GPXGXGKT), motif C (FDKDRGXE), Walker B (RIXXXXDGXXXXXXXDE), motif D (LXXXRKXN), motif E (IXATQ)	(103, 105)
VirB4-2	Walker A (GXXXXG**K/R**), Walker B (SLXXXXXXXIXXXDX)	
VirB6-1	Variable TM region (VXAFXXLYXXXXGXXILLX), conserved cytoplasmic loop (PXXXXXXXFXXTXXXXXXW)	(105–107)
VirB6-2	Variable TM region (XXAALXLYXXFFXXXXXXX), conserved cytoplasmic loop (PXXXXXXXFXXTXXXXXXW)	
VirB6-3	Variable TM region (IXAXLXLYXMXXGXXFXLG), conserved cytoplasmic loop (PXXXXXXXFXXTXXXXXXW)	
VirB6-4	Variable TM region (XXXXXXLYXTXXGXXFXLG), conserved cytoplasmic loop (PXXXXXXXFXX**T/I**XXXXXXW)	
VirB6-5	Variable TM region (VXXXLXLXXXFXGXXFLIG), conserved cytoplasmic loop (PXXXXXXXFXXTXXXXXXW)	
VirB7	Conserved cys between positions 15–35, possibility of being a small lipoprotein	(103)
VirB8-1	Homodimerization domain I (YXXXREXY), VirB4 interaction region (XXXY**K**)	(103, 108, 109)
VirB8-2	Homodimerization domain I (YXXXREXY), NPxG motif (NPXG), VirB4 interaction region (XXXY**R**)	
VirB9-1	DXR-YXP motif (DXRXXXXXYXP), Beta 1 (NXXYXX), Beta 2–3-OM extrusion region (PXXXXDXXXXTXXXF - PXXXXX**G/DD**XXXE), VirB7 interaction region (RXGXXXXCXXN)	(103)
VirB9-2	DXR-YXP motif (DXRXXXXXYXP)	
VirB10	OM pore gating (DXLGXXGXXGXV), Beta 6 a (VLXSAX), Beta 7a (XTXXXNQG)	(105, 110)
VirB11	Linker A (IRXXSXXXXXL), Beta 1 (XXEXXXNXPG), Beta 5 (LPXXXRXQXXXPP), ATPase region/Beta 7-Alpha E (GXTXXXKTT), Beta 8 (ERXIXXED), Alpha F-Beta 10 (LXXXXLRXRPDRIXXXE), Beta 11 (GHPGSIXTXH)	(111)
VirD4	DNA-binding motif A (APTXXGKGXGXVIPXXXXXXXSVXXXDXK), DNA-binding motif B (XFLLDEFXXLGKXXX)	(112, 113)

^*a*^ Underlining indicates important amino acids in the motif.

^*b*^ Boldface indicates variability in amino acid sequence.

VirB1 and VirB5 could not be identified in Ot. However, VirB7 was annotated based on gene positioning, conserved cysteine(s), and its important role of being a small lipoprotein in T4SS of *Rickettsia* species.

These *rvh* genes identified in Boryong were then set as a reference for other Ot strains. Each *vir* gene was then blasted (nucleotide blast using Geneious Prime) to identify the presence of each *rvh* across the eight strains of Ot (Boryong, Ikeda, Karp, Kato, Gilliam, TA686, UT76, and UT176). The amino acid sequences of each Rvh subunit from the eight strains of *Ot* were then aligned (multiple alignment, Clustal Omega) to verify their motifs. Even though some of the gene copies appear to be a truncation or pseudogene due to loss of some motif(s) like that in RvhB4-II, RvhB8-II, and RvhB9-II, they are well characterized in literature. So, these names were kept the same in our annotation.

### Analysis of F-type IV secretion systems (RAGE T4SS)

Literature search and blast search (NCBI and KEGG) were performed to identify the presence of each Tra subunit (F-type T4SS: TraA to TraN, TraU to TraW, TrbC, and TrbE; P-type T4SS: TraA_Ti_ and TraD_Ti_) in Ot. The amino acid sequences of each Tra subunit present in Ot Ikeda strain (OTT) were then compared to their respective orthologs in RBE and *Escherichia coli* (ECO) for F-type T4SS or ATU for P-type T4SS. The presence of motifs was used as the major criteria to identify Tra subunits as indicated in Table 4.

**TABLE 4:** Motifs conserved in Tra and Trb proteins

Subunit	Motif(s)	References
TraE	Anchor region (**LVKY**NKXLLXXTXXL/IAXXXX), predicted conserved region 1 (SXXXXXXXYLXXXA), predicted conserved region 2 (KXXXXXSXFFXXXXXV), predicted conserved region 3 (VXIXGXXXXWXXXXKXXXXX**K/R**XYXLXXK)-GenomeNet Motif search (TraE region)	(114–116)
TraB	Coiled-coil domain (IX**XXXQ/K**XXXX**L/F**XXXXKXXXX), predicted conserved region (GXXSSERAXXR)-GenomeNet Motif search (TrbI-like region), OM pore gating (GXXGXXGXV), Alpha-3 (GXXXGXXX**A/V**XXXLXDXXIK**R/Q**AXXXXP)	(117, 118)
TraV	Conserved cysteine region (C**X**XXXXXXXXXXX**F/L/V**XCXXXXXXXC), predicted conserved region (LXXL**F/L**XXXXXX**G/C**E)-**GenomeNet Motif search (TraV region)**^*a*^	(119, 120)
TraC	Predicted conserved region 1 (YXXYXX**E/K**XXLFXNXXXXGFXLXXXP), predicted conserved region 2 (YXXLXXQXXXXXFXLXXXXD)-GenomeNet Motif search (TraC region), Walker A (GXXGXGKX), Motif C (**V/A**XDXGXXXK), Walker B (RXXXXLXXIDEXW), motif D-E (RXXXGXXXXXTQ)^*b*^	(118, 121, 122)
TraW	TrbC interaction region (EXXXLXVIMXXLXXXXXXGXXXXXXXXF), predicted conserved region 1 (N**P/S**LXXXXXXXXLXXIXGDDXXQVXWXK), predicted conserved region 2 (FDQXXXLXXXXXIXXXPA)-InterPro Motif search (TraW region)	(123)
TraU	Signaling domain (AXXXCXG), Hydrophobic-2 (CMVXL**G/W**), Hydrophobic-3 (YWLXIXX), Hydrophobic-4 (FXNXXAXXACXAD), Hydrophilic-1 (KXXXRXQM), Hydrophilic-2 (**W/L**RKRX**C/Y**)	(114, 124)
TrbC	Signaling domain (MXIRVMXLXXLLXVNN), predicted conserved region 1 (FVSFSXXXXXLK), predicted conserved region 2 (GXXXXXR**G/R**XXNNXXXXT)-GenomeNet Motif search (TrbC region), TraW interaction region (ID**P/S**XLFXXYXXXXV**P****/L**XXVX)	(123, 125)
TraN	Conserved cysteine region 1 (SCXEGXX), conserved cysteine region 2 (SXCXXXE), conserved cysteine region 3 (IGXXC), conserved cysteine region 4–5 (CXXXKXXYCXFXS**K/R**LAXXX**Q/H**), conserved cysteine region 6 (CR**G/D**XTVX**E/K**LQXXXF), predicted conserved region-1 (ECX**E**), Predicted conserved region 2 (CXLXXXXC), predicted conserved region 3 (CLXXXXXYX**C**), predicted conserved region 4 (CXKXXXXXX**N/H**CC)-GenomeNet Motif search (TraN region)	(126)
TraF	Predicted conserved region (**G/X**XXWYNX)-GenomeNet motif search (TraF region), C-X-X-C motif (CXXC), Beta 10 (**V**PXXX**L/S**X), Alpha 7 (ISX**D/N/E**XXXXXXL)	(127)
TraH	TrbI interaction region 1 (TXXGXXQX**Q/L**AAGYYXXGXLXXRT), TrbI interaction region (NIXXXAX), predicted conserved region 1 (CXXIXXYLXSFSXIX**G/V/RE**XL), predicted conserved region 2 (FLSSIGXXXXXXXYXXXXXISG), predicted conserved region 3 (LXQXXXEXXXXXR)-GenomeNet motif search (TraH)	(122, 128)
TraG	Membrane spanning region 1 (**M/W**XWXXXXXXIXXXLXXX), membrane spanning region 2 (QSVXXX**L/V**XXXXXXXVFPMXXLXXXXXIXKXWIXXIIWVXSWPVXF), membrane spanning region 3 (XX**A/ST/M**XXXLAXXXPXLSWXVX**K/N**XXXXXXXXLXXXFSXXXV), cleavage site (ASXXGX), predicted conserved region 1 (SXXXXLSXXL), predicted conserved region 2 (KQXXEQXXXXXXYXXQXS)-GenomeNet motif search (TraG N-terminal region)	(129)
TraD	Transmembrane region 1 (MXXQXXXNXXXIGLXXXXXWXXXXXYQ), transmembrane region 2 (FLXXSXXXEXXXXFXIX), Walker A (GTXGXGKXX), Walker B (XXWFXXDELP)	(114, 130)
TraI	Helicase region 5 (XHGYAXTXXXX**Q/K**XAXXXXXXVLXXXXXXXXX), predicted conserved region 1 (IXEGXEXXXXLXXXXIXGXIIXXXX**I/V**XXXXNXX**P/L**XX**G/S**), predicted conserved region 2 (AVXNXVXXXXAXXVX**E/D**XKXXXXXXXKXXFNXVLKXX**GL**)-GenomeNet motif search (Toprim region)	(131)
TraA_Ti_	Nickase region 1 (AIXFXXXXXXXR**S/I**XGXXSCX**K/N**XXYXXXXXXXXXXXXXXXXXXXXXXXVXH), Nickase region 2 (NEVE**R/Q**XXX**X**XXNSXXXXXIVI**A/V**L**P/Q**), Nickase region 3 (NXHX**H/N**XXXXXRXXXXXG), helicase region 1 (XXGXAGXGKXXXXXX**A/V**), helicase region 2 (XX**V/I**XD**E/K**AGM**V/A**), helicase region 3 (XXXXXLXGDXXQ**L/R**XXXEXGXXFXXXXXXXXXXXL), helicase region 5 (XHGYAXTXXKX**Q/H**GAXXXXXX**V/I**LXXXXXXXXX), helicase region 6 (Y**V/T**XM**T/I**R**H/Y**XXXXXLY)	(131, 132)
TraD_Ti_	Predicted conserved region 1 (RKXXX**R/Q**XXXXX**G/A**XX**V/L**XXAXL), predicted conserved region 2 (IGXXXFXXXXXN)-GenomeNet motif search (TraD region)	(131)

^*a*^ Underlining indicates important amino acids in the motif.

^*b*^ Boldface indicates variability in amino acid sequence.

Note that TraA_Ti_ found in *Rickettsia* is fragmented into TraA_Ti_ and TraI in *Ot*. The longest *tra* and *trb* genes identified in Ikeda were set as references and were then blasted (nucleotide blast using Geneious Prime) to identify their presence across the eight strains of Ot (Boryong, Ikeda, Karp, Kato, Gilliam, TA686, UT76, and UT176). The amino acid sequences of each Tra subunit from the eight strains of Ot were then aligned (multiple alignment, Clustal Omega) to verify their motifs. Those amino acid sequences with difference greater than 10% from full-length gene in Ikeda or missing motif(s) were classified as pseudogenes.

## Data Availability

All data generated by this work are available within the article and supporting information.

## SUPPLEMENTAL MATERIAL

Data Set S1 msphere.00268-23-s0001.xlsx Genomes.

Data Set S2 msphere.00268-23-s0002.xlsx Membrane proteins.

Data Set S3 msphere.00268-23-s0003.xlsx MRP and HK.

Data Set S4 msphere.00268-23-s0004.xlsx Hypothetical proteins.

Data Set S5 msphere.00268-23-s0005.xlsx Ankyrin repeat proteins.

Data Set S6 msphere.00268-23-s0006.xlsx Tetratricopeptide repeat-containing proteins.

Data Set S7 msphere.00268-23-s0007.xlsx Gene transfer agents.

Data Set S8 msphere.00268-23-s0008.xlsx Type 4 secretion systems.

Supplemental Figures msphere.00268-23-s0009.pdf Figures S1 to S3.

Captions msphere.00268-23-s0010.docx Captions for Data Sets S1 to S8.

ASM does not own the copyrights to Supplemental Material that may be linked to, or accessed through, an article. The authors have granted ASM a non-exclusive, world-wide license to publish the Supplemental Material files. Please contact the corresponding author directly for reuse.
