# Supplementary material for: Orientia tsutsugamushi: comprehensive analysis of the mobilome of a highly fragmented and repetitive genome reveals the capacity for ongoing lateral gene transfer in an obligate intracellular bacterium
Source: mSphere. 2023 Oct 18;8(6):e00268-23. doi: 10.1128/msphere.00268-23 (PMC10732058; doi:10.1128/msphere.00268-23)
Supplement: Supplemental Figures — Figures S1 to S3. [file msphere.00268-23-s0009.pdf]

Orientia tsutsugamushi: analysis of the mobilome of a highly fragmented and repetitive genome reveals ongoing lateral gene transfer in an obligate intracellular bacterium.

Suparat Giengkam[a], Chitrasak Kullapanich[a], Jantana Wongsantichon[a], Haley E. Adcox[b], Joseph J. Gillespie[c] and Jeanne Salje[a, d]

Mahidol-Oxford Tropical Medicine Research Unit, Faculty of Tropical Medicine, Mahidol University, Bangkok, Thailand[a] ;

Department of Microbiology and Immunology, Virginia Commonwealth University Medical Center, School of Medicine, Richmond, Virginia, USA[b];

Department of Microbiology and Immunology, School of Medicine, University of Maryland Baltimore, MD 21201[c];

Department of Biochemistry/Department of Pathology/Cambridge Institute of Medical Research, University of Cambridge, UK[d];

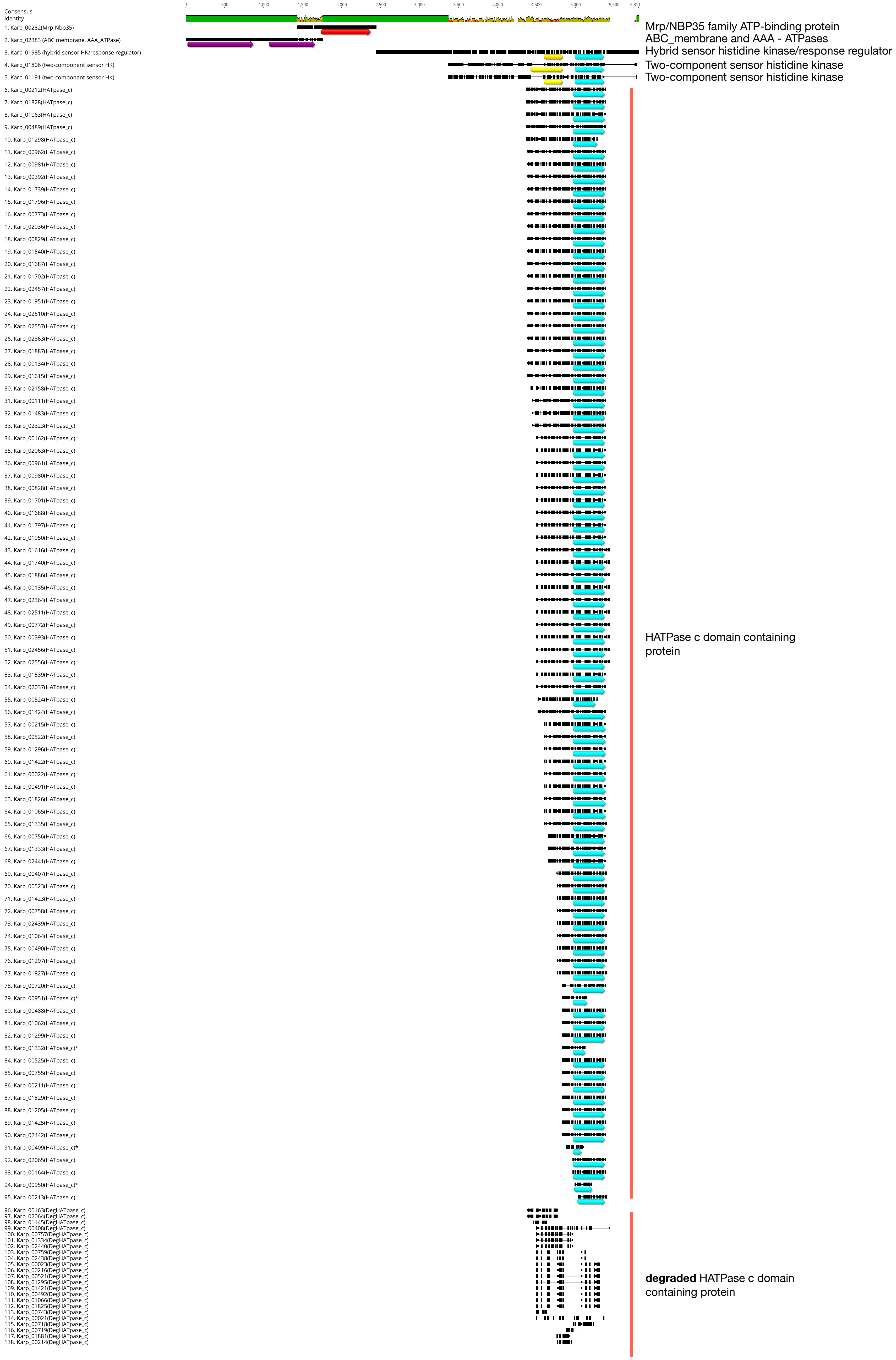

Supp. Fig. 1. Alignment of MRP and HATPase-domain genes in Ot Karp.

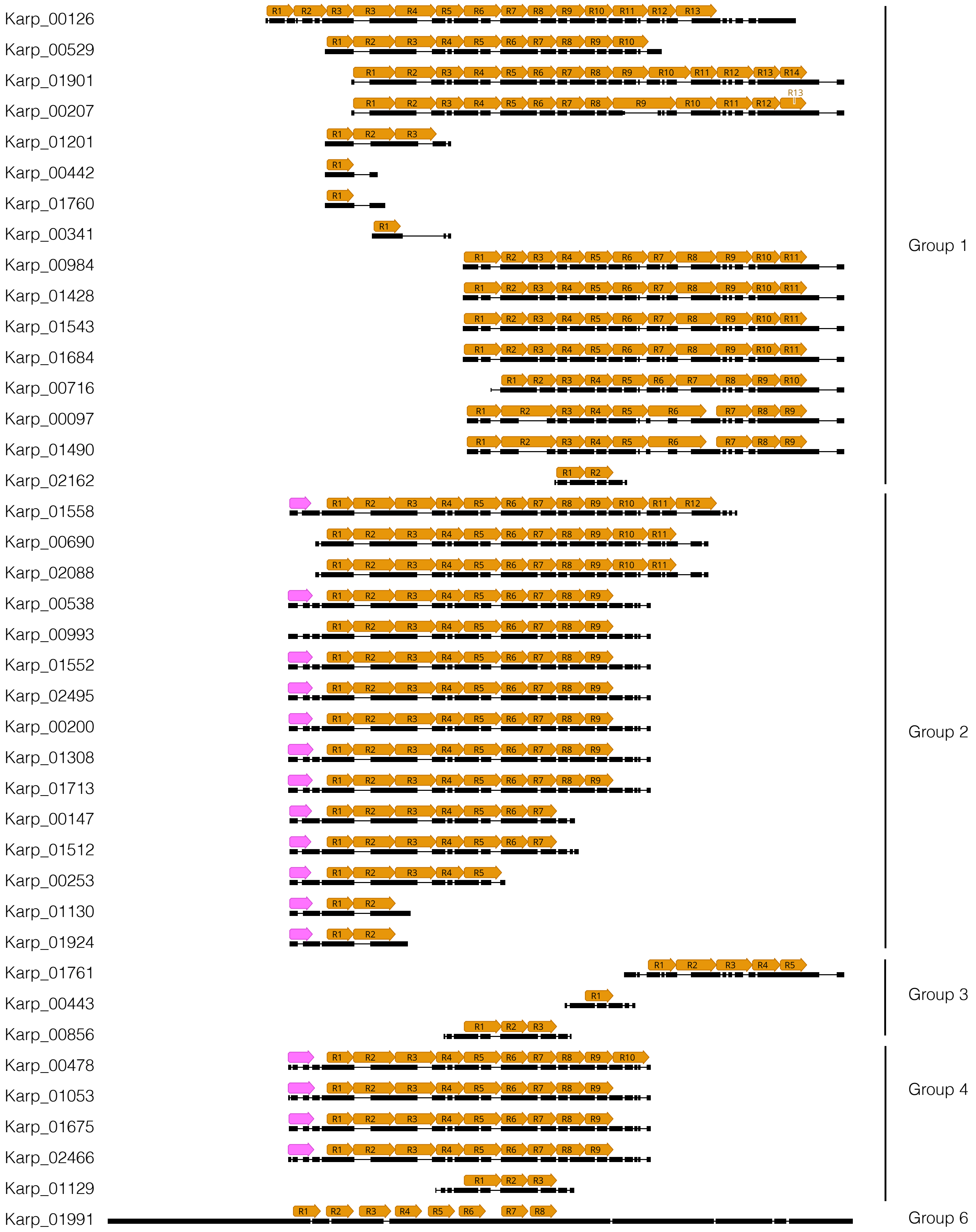

Supp. Fig. 2. Analysis of tetratricopeptide repeat containing proteins in Ot (TPRs). A. Alignment of TPR genes in Ot strain Karp showing classification into groups.

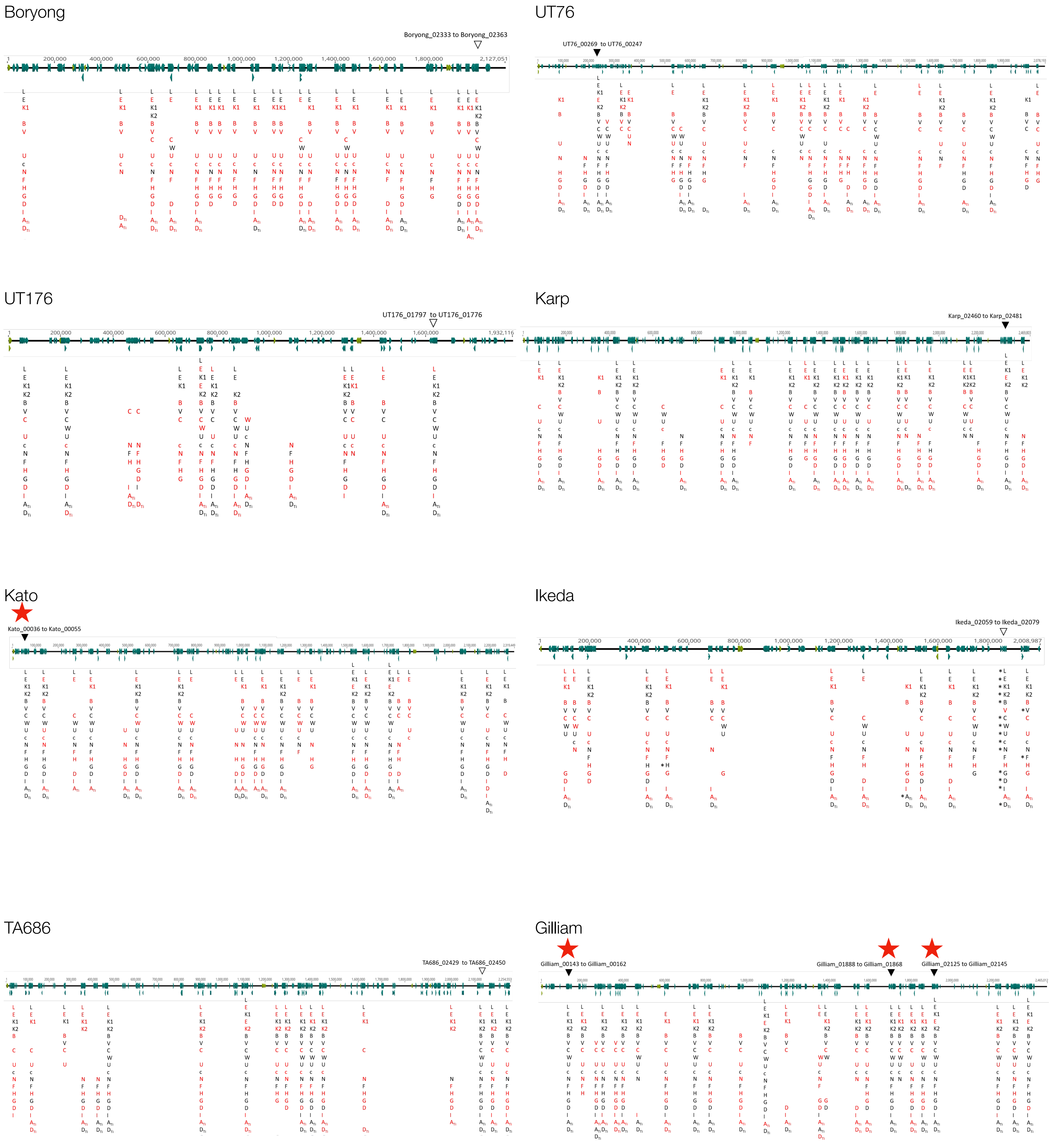

Supp. Fig. 3. Analysis of Tra genes in Ot. An overview of *tra* gene clusters in all Ot strains. Truncated genes are shown in red, and full length tones in black. Complete gene sets are shown in black arrows above, with almost complete sets in white arrows. Full gene sets located within complete RAGEs are shown by red stars.
