# Supplementary material for: Orientia tsutsugamushi: comprehensive analysis of the mobilome of a highly fragmented and repetitive genome reveals the capacity for ongoing lateral gene transfer in an obligate intracellular bacterium
Source: mSphere. 2023 Oct 18;8(6):e00268-23. doi: 10.1128/msphere.00268-23 (PMC10732058; doi:10.1128/msphere.00268-23)
Supplement: Captions — for Data Sets S1 to S8. [file msphere.00268-23-s0010.docx]

**Supplementary Dataset 1: Genomes**

An overview of the eight complete Ot genomes analysed in this study: Gilliam, Boryong, UT76, UT176, Karp, Kato, Ikeda, TA686. Each tab contains genome annotation data for one strain. Genes are classified as being in RAGE or IR regions, or annotated as isolated hypothetical proteins, isolated cargo genes, or isolated mobile genes (column B). IR regions are named consistently across all genomes. In some cases, IR gene groups are intact in some genomes but split between two positions in other genomes. In these cases this information is provided in column B. New gene name annotations, and analysis of full-length/truncated or degraded are given in columns C and D. RNAseq and proteomics data (Karp)(1) and proteomics data (UT76)(2) from previous studies are included in those strains.

1. B. Mika-Gospodorz *et al.*, Dual RNA-seq of Orientia tsutsugamushi informs on host-pathogen interactions for this neglected intracellular human pathogen. *Nat Commun* **11**, 3363 (2020).

2. S. Atwal *et al.*, The obligate intracellular bacterium Orientia tsutsugamushi differentiates into a developmentally distinct extracellular state. *Nat Commun* **13**, 3603 (2022).

**Supplementary Dataset 2: Membrane proteins**

An overview of the analysis of genes annotated as membrane proteins in Gilliam, Boryong, UT76, UT176, Karp, Kato, Ikeda and TA686. A summary tab provides an overview of the classification of the membrane proteins, whilst individual tabs represent analyses for individual strains.

**Supplementary Dataset 3: MRP and HK**

An overview of the analysis of multidrug resistance proteins (MRP) and histidine kinase domain (HK) genes in Gilliam, Boryong, UT76, UT176, Karp, Kato, Ikeda and TA686. Summary tabs provide overview of methods of analysis, and overall findings, whilst individual tabs represent analyses for individual strains.

**Supplementary Dataset 4: HPs**

An overview of the analysis of hypothetical proteins (HP) in Gilliam, Boryong, UT76, UT176, Karp, Kato, Ikeda and TA686. All genes previously annotated as hypothetical or uncharacterized are included in this dataset. Summary tabs provide overview of methods of analysis, and overall findings, whilst individual tabs represent analyses for individual strains. Yellow= HP is located in RAGE region, Blue= HP is located in IR-region, Gray= Isolated HP

**Supplementary Dataset 5: Anks**

An overview of the analysis of Ankyrin repeat containing proteins (Anks) in Gilliam, Boryong, UT76, UT176, Karp, Kato, Ikeda and TA686. A summary tab presents an overview of all the Anks in all the strains, whilst individual tabs give analyses for separate strains.

**Supplementary Dataset 6: TPRs**

An overview of the analysis of tetratricopeptide repeat containing proteins (TPRs) in Gilliam, Boryong, UT76, UT176, Karp, Kato, Ikeda and TA686. A summary tab presents an overview of all the TPRs in all the strains, whilst individual tabs give analyses for separate strains.

**Supplementary Dataset 7: GTAs**

Identification of GTA genes in Ot genomes, identified through BLAST homology to GTA genes in Wolbachia. The geneIDs of the Ot genomes and the Wolbachia genes are provided.

**Supplementary Dataset 8: T4SS**

An overview of the analysis of RAGE F-type 4 SS (F-T4SS) in Gilliam, Boryong, UT76, UT176, Karp, Kato, Ikeda and TA686. A summary tab presents an overview of the presence of tra genes in all the Ot genomes, whilst individual tabs give analyses for separate strains.
